# Immunomodulatory Nanomaterials: Design Strategies, Mechanisms, Biomedical Applications, and Future Perspectives

**DOI:** 10.3390/biomedicines14050964

**Published:** 2026-04-23

**Authors:** Maharshi Thalla, Sumedha Kapre, Sushesh Srivatsa Palakurthi, Praveen Kolimi, Ravi Akkireddy, Geetha Satya Sainaga Jyothi Vaskuri, Nagavendra Kommineni, Rahul Sharma, Jae D. Kim, Srinath Palakurthi

**Affiliations:** 1 Department of Pharmaceutical Sciences, Irma Lerma Rangel College of Pharmacy, Texas A&M University, Kingsville, TX 78363, USA; 2 Department of Pharmaceutics and Drug Delivery, School of Pharmacy, University of Mississippi, Oxford, MS 38677, USA; 3 Cancer Center, School of Medicine, Texas Tech University Health Sciences Center, Lubbock, TX 79430, USA; 4 Department of Pharmaceutical Sciences, University of Tennessee Health Science Center, Memphis, TN 38163, USA; 5 Thermo Fischer Scientific Inc., Cincinnati, OH 45237, USA; 6 Division of Nephrology, Department of Medicine, University of Virginia, P.O. Box 800133, Charlottesville, VA 22903, USA; 7 Corpus Christi Gastroenterology PLLC (Professional Limited Liability Company), Saratoga Boulevard Building, Corpus Christi, TX 78414, USA

**Keywords:** nanomaterials, immunomodulation, immune regulation, disease treatment, wound healing and bone regeneration

## Abstract

The utilization of immunomodulatory nanomaterials, i.e., leveraging their unique properties to enhance immune responses, represents a transformative approach for the treatment of various diseases. Recent advancements in nanotechnology have enabled the design of nanomaterials capable of delivering immunomodulatory agents in a targeted manner, such as cytokines, antibodies, and nucleic acids, to specific cells or tissues involved in immune regulation. These nanomaterials, including nanoparticles, liposomes, nanogels, nanoemulsions, dendrimers, MXenes and extracellular vesicles, have been increasingly tailored to modulate immune responses with precision and efficacy. This targeted approach not only enhances therapeutic outcomes but also reduces off-target effects, minimizing systemic toxicity. In this review, an overview of immunomodulatory nanomaterials and their biomedical applications are highlighted. Herein, we have discussed different types of nanomaterials and their design strategies, interactions with different immune system components (macrophages, dendritic cells (DCs), neutrophils, T lymphocytes (CD4^+^ helper T-cells, CD8^+^ cytotoxic T-cells, regulatory T-cells/Tregs, and memory T-cells), and B lymphocytes), and immunomodulation mechanisms. Furthermore, nanomaterial-based immunomodulation strategies to enhance cancer immunotherapy, wound healing, and bone regeneration and the treatment of infectious diseases, autoimmune diseases, and allergy and are discussed in detail. In addition to therapeutic applications, selected nanomaterial platforms demonstrate significant potential in pharmaceutical formulations by improving drug stability, controlled release, and bioavailability, as well as in cosmetology through skin-targeted delivery, anti-inflammatory activity, immune protection, and enhanced tissue regeneration. Finally, clinical trial updates, challenges and future prospects are outlined. Key findings indicate that lipid-based, polymeric, inorganic nanoparticles and dendrimers provide complementary advantages for immunomodulation, including efficient delivery, controlled release, multifunctionality, and precise immune targeting. Despite safety, regulatory, and scalability challenges, these systems show strong potential for advancing precision and personalized medicine. Taken together, these innovations hold great promise for personalized medicine approaches, wherein nanomaterials can be tailored to individual patient profiles for more effective and precise disease treatment and prevention strategies. This review focuses primarily on the mechanistic interactions between immunomodulatory nanomaterials and immune cells, including macrophages, dendritic cells, neutrophils, T lymphocytes, and B lymphocytes, rather than providing an exhaustive treatment of physicochemical optimization parameters such as particle size or surface modification chemistry, which fall outside the defined scope of this work.

## 1. Introduction

The immune system plays a central role in protecting us from a variety of illnesses and diseases through the maintenance of tissue homeostasis. Additionally, a wide range of disease processes are mediated by different immune responses [1]. Dysregulated immune responses can result in various health issues, such as cancer, infections, autoimmune disorders, and the rejection of transplants [2,3,4,5]. To properly control and prevent immune-related disorders, the immune environment is regulated by several immune cells, enzymes, and cytokines. For example, a highly immunosuppressive state is maintained in the case of cancer via the recruitment of immunosuppressive immune cells and the expression of various inhibitory cytokines, checkpoint molecules, and enzymes, which facilitates both tumor evasion from immune surveillance and the progression of carcinogenesis by creating a pro-tumorigenic microenvironment [6]. In contrast, a series of inflammatory activities are elicited by the overactive immune system in the case of autoimmune syndromes and inflammatory diseases [7]. Currently, various upstream and downstream approaches have been employed as standard therapeutic interventions. Upstream approaches utilize the systemic administration of wide-spectrum immunosuppressive drugs, which are limited by systemic side effects, rapid clearance, opportunistic infections and malignancy [8]. On the other hand, downstream approaches employ the interactions of specific targets with anti-inflammatory agents. This approach elicits weak inhibitory effects in the complex and heterogeneous inflammatory network [9,10]. Other factors that negatively affect immunotherapeutic methods include poor solubility, loss of bioactivity after long circulation, and high immune-mediated toxicity [11]. Moreover, these standard therapies are palliative and provide only short-term relief through the use of anti-inflammatory and immunosuppressive agents.

Traditional drug delivery techniques, such as administering small-molecule drugs orally or intravenously, have a number of intrinsic drawbacks. Poor drug solubility, low bioavailability, rapid disintegration, non-specific distribution, and the incapacity to target particular immune cells or sick tissues are some of them. These limitations affect long-term clinical outcomes by increasing systemic toxicity and side effects in addition to decreasing therapeutic efficacy. Additionally, traditional formulations frequently fall short of achieving sustained or regulated release, which results in inconsistent drug levels and inadequate immunomodulation [12]. Therefore, there is an utmost requirement for alternative strategies to further improve the management of hyperactive/underactive immunity-related disorders while overcoming the drawbacks of standard therapies.

Recent advancements in the nanotechnology and bioengineering research domain over the last decade have opened new avenues for the development of various emerging strategies for immunoregulation [1,13,14,15]. Nanomedicine-based precision interventions have greatly improved the safety and effectiveness of anti-inflammation therapies and immunoregulation. Nanomaterial-mediated immunoregulation considerably reduces the off-target effects and systemic toxicity of anti-inflammatory agents or immunosuppressants [16]. Furthermore, nanomaterials can be engineered for the diagnosis and therapy of human disease. These engineered nanomaterials either suppress or stimulate the immune system in a targeted manner [17,18,19]. The physicochemical properties of nanomaterials, such as their particle size, shape, hydrophobicity, stiffness, and surface components, are tailored to induce interaction with immune cells. The advantages of engineered nanomaterials include on-demand payload release, efficient drug encapsulation and targeting, improved pharmacokinetic profiles, enhanced transport across membranes, and increased safety and/or efficacy [20,21,22]. Examples of FDA-approved or preclinically promising engineered nanomaterials include organic or inorganic-based nanoparticles (NPs), polymeric nanoparticles, lipid-based nanoparticles, nanogels, nanoemulsions, nanocapsules, dendrimers, etc. [16,23,24,25,26].

The emerging concept of immunomodulatory nanosystems (IMNs) using a combination of nanomaterials and immunotherapy can readily improve therapeutic outcomes by quickly overcoming the shortcomings of earlier treatment methods [16,27,28,29]. Nanomaterial-induced immunostimulation or immunosuppression mediates immunotherapy by affecting the activity and function of immune cells [30,31]. The nanoscale size immunomodulatory nanosystems efficiently modulate the immune system by interacting with different immune cells, such as inducing the expansion of regulatory T (Treg) cells, modulating macrophage polarization, interacting with receptors on cell surfaces, or generating tolerogenic dendritic cells, for significant therapeutic benefits [29,32,33,34,35,36]. Immunomodulatory nanomaterials enhance immune system function by effectively escalating the activation of antigen-presenting cells (APCs) and preventing the degradation of antigens by enzymes inside the body through coating or conjugating with antigens and adjuvants. Moreover, antigen–adjuvant complexes in circulation are stabilized by immunomodulatory nanomaterials for sustained release of antigens to target sites [28,37]. Immunomodulation using nanomaterials has garnered significant attention for application in regressing cancer, tuberculosis, viral infections and other infectious diseases [16,23,24,27,38]. Earlier reports demonstrated activation of pro-inflammatory macrophage polarization using FDA-approved iron oxide nanoparticles (IONPs) (i.e., ferumoxytol) for suppression of cancer growth [39,40]. In other studies, nanomaterial-based activation of the immune system for therapeutic interventions for different autoimmune, inflammatory and infectious diseases has been reported and has demonstrated positive outcomes [16,23,27,38].

Taken together, a deeper understanding of nanomaterial-based immunomodulation towards improving immunotherapeutic efficiency is pertinent. Against this background, we reviewed the recent advancements and future prospects of immunomodulatory nanomaterials for therapeutic interventions. Herein, we highlight the different types of nanomaterials for immune system modulation (stimulation/immunosuppression) followed by direct and indirect mechanisms depicting their interactions with the immune system. We have also outlined the interactions of nanomaterials with specific immune cells such as macrophages, neutrophils, and lymphocytes (B-cells and T-cells) for the treatment of different inflammatory and infectious diseases. Furthermore, biomedical applications of nanomaterial-based immunomodulation strategies to enhance cancer immunotherapy, the treatment of autoimmune diseases, tuberculosis and other infectious diseases are specifically discussed. Finally, the clinical trial updates, challenges and future prospects for immunomodulatory nanomaterials are also predicted.

### Literature Search Strategy

The literature search for this narrative review was conducted using the PubMed, Scopus, Web of Science, and Google Scholar databases. Searches were performed for articles published primarily between 2015 and 2025, with seminal earlier works included where contextually appropriate. The following keyword combinations were used: “immunomodulatory nanomaterials,” “nanoparticles immune cells,” “nanoparticles macrophage polarization,” “polymeric nanoparticles immunomodulation,” “lipid nanoparticles immune response,” “dendritic cells nanoparticles,” “regulatory T-cells nanoparticles,” “Tregs nanoparticles,” “B-cells nanomaterials,” “neutrophils nanoparticles,” “nanomaterials cancer immunotherapy,” “nanomaterials autoimmune disease,” “nanomaterials infectious disease,” “nanomaterials wound healing,” “nanomaterials bone regeneration,” and “clinical trials immunomodulatory nanomaterials.” Articles were selected based on relevance to the mechanistic, therapeutic, and translational aspects covered in this review. The scope was deliberately focused on the mechanistic interactions between nanomaterials and immune cells and their therapeutic applications.

## 2. Types of Nanomaterials Utilized for Immunomodulation and Their Design Strategies

Nanomaterials designed for immunomodulation provide a versatile and powerful approach to modulating the immune system for therapeutic use. By accurately targeting and managing immune responses, these nanomaterials could transform treatments for cancer, autoimmune disorders, infectious diseases, and organ transplants, as depicted in Table 1. The precise interactions of nanomaterials with the immune system, achieved through either the enhancement or suppression of immune responses, have been utilized for immune modulation. Nanomaterials can act as immunomodulators in two ways; direct or indirect [41]. The direct approach involves direct interaction between nanoparticles and immunological responses, whereas the indirect approach involves the use of nanomaterials as carriers of immunomodulatory drugs [41]. Lipid, gold, polymeric, and silica nanoparticles, virus-like particles, nanogels, and extracellular vesicles are a few examples that are utilized in inflammatory, autoimmune, infectious, and cancerous disorders [23,24,42].

Furthermore, strategic modulation of the immune response through nanomaterials holds immense potential for advancing therapies across a range of diseases. The strategies include surface functionalization, controlled release mechanisms, size and shape optimization, multifunctionality, and biocompatibility [1,43]. Nanomaterials offer remarkable therapeutic promise for autoimmune, infectious, inflammatory, and cancerous disorders by enabling precise, stimuli-responsive regulation of immune cells such as dendritic cells, macrophages, T-cells, and B-cells [43]. A representative list of different nanomaterials being utilized for immunomodulation along with their design strategies is shown in Table 2. Moreover, advanced nanomaterials with tailored properties may be developed for specific therapeutic needs. For clinical translation to progress and overcome the present obstacles, further research is essential.

A wide range of NPs, including organic nanomaterials (e.g., polymeric NPs, lipid NPs, liposomes, nanostructured lipid carriers (NLCs), polymeric micelles), inorganic nanomaterials (e.g., metal-based NPs (gold, manganese, zinc and iron)), silicon-based NPs, quantum dots, and carbon-based NPs, have been utilized as immunomodulatory systems [44]. Organic and inorganic nanoparticles play a pivotal role in the field of immunomodulation due to their unique properties and functionalization capabilities. Both organic and inorganic nanoparticles offer unique advantages for immunomodulation, with each type suitable for different therapeutic applications and contributing to clinical translation [45].

**Table 1:** A representative list of different immunomodulatory nanomaterials, their immune response, and applications in disease treatment.

Nanomaterial Type		Immune Response Type	Immune System Modulation	Diseases	Findings	References
Organic nanomaterials	Virus-like particles	Adaptive immune response	Stimulation	Cancer	Efficient B-cell receptor crosslinking, strong humoral response with IgG antibody production, rapid lymph node accumulation (10 min), effective cross-presentation via MHC I/II pathways	[42]
	Liposomes	Adaptive and innate	Suppression	Cancer, autoimmune diseases	Decreased cytokine production by macrophages and CTLs, reduced tumor-infiltrating T-cells	[46,47,48,49]
	Polymeric nanoparticles	Adaptive immune response	Stimulation	Infectious diseases	Enhanced antigen-specific IgG responses in immunized mice, 95% complexation efficiency, induces strong adjuvant effects with neutralizing antibody production	[27,28]
	Poly (lactic-co-glycolic acid) (PLGA) nanoparticles	Adaptive immune response	Stimulation	Cancer, infectious diseases, autoimmune diseases, drug delivery	Increased splenic CD8^+^ T-cells (26.8% vs. 18.9%), B-cells (83.1% vs. 59.5%), and tumor-infiltrating macrophages (18.2% vs. 11.7%)	[50,51,52,53]
	Polyethyleneimine nanoparticles	Adaptive immune response	Suppression	Cancer	Targets cancer cells, stimulates immune response, activates TLR4-mediated immune responses with minimal cytotoxicity < 10 μg/mL	[54,55]
Inorganic nanomaterials	Gold nanoparticles	Innate immune response	Stimulation	Cancer, inflammatory diseases	Strong immune response with high antibody titers and IFN-γ production, enhanced T-cell responses	[28,56,57,58,59,60]
	Carbon nanotubes	Adaptive and innate	Stimulation	Cancer, autoimmune diseases	Activated immune-related pathways (IL-6, CD40, DC maturation, TNF-α/TNFR, NF-κB, Th1 chemokines CXCR3/CCR5), greater immunomodulatory potency with IL-1β secretion	[61,62,63,64]
	Magnetic nanoparticles	Adaptive and innate	Stimulation	Cancer	Facilitates targeted hyperthermia, inducing immunogenic cell death and systemic anti-tumor immune response, M2-to-M1 macrophage repolarization, and magnetic field guidance, enhances T-cell migration to tumor sites, activates immune cells by cytokine production	[65,66,67]
	Silica nanoparticles	Adaptive immune response	Stimulation	Cancer, infectious diseases	Induced IL-1β and IL-8 production via inflammasome formation, increased regulatory T-cell subsets (CD25^+^), supported dendritic cell antigen-presenting properties	[68,69,70,71]
	Calcium phosphate nanoparticles	Adaptive immune response	Stimulation	Infectious diseases	Enhanced antigen uptake by DCs via increased CD80/CD86 expression and IL-6 production	[72]
	Iron Oxide nanoparticles	Innate immune response	Stimulation	Cancer, allergic diseases, infectious diseases	Induced M2-to-M1 macrophage repolarization, significantly enhanced tumor infiltration of DCs (18.2% vs. 11.7%) and activated CD8^+^ T-cells (CD69^+^ CTLs)	[39,73,74]
	Quantum dots	Adaptive and innate	Stimulation	Cancer, autoimmune diseases	Induced immune tolerance, targeted therapy	[75,76]
	Zinc oxide nanoparticles	Innate immune response	Stimulation	Cancer, infectious diseases	Antibacterial properties, induced apoptosis in cancer cells	[77]
	Silver nanoparticles	Innate immune response	Suppression	Infectious diseases	Reduced IL-6 mRNA while increasing TGF-β1 during wound healing, immunosuppressive without major cytotoxicity	[77,78,79]
	Mesoporous silica nanoparticles	Adaptive immune response	Stimulation	Infectious diseases, cancer	Enhanced H7-specific humoral and Th1/Th17-polarized T-cell responses, efficient DC uptake into endosomal compartment with increased TNF-α, IL-6, IL-12 production	[68,70,71,80]
	Titanium dioxide nanoparticles	Innate immune response	Stimulation	Cancer	Photocatalytic properties, induces immune response, induces dose-dependent pro-inflammatory responses with increased TNF-α, IL-6, MIP-2, IP-10, MCP-1	[77]
Nanogels	Nanogels	Adaptive immune response	Stimulation	Cancer, infectious diseases	Promoted immune cell infiltration, stimulated humoral immune response, controlled antigen release kinetics	[60,81,82,83]
	Polysaccharide nanogels	Innate immune response	Suppression	Drug delivery, infectious diseases	Enhanced CD40^+^ and CD86^+^ expression, promoted controlled antigen release and lymph node translocation enhanced mucosal immunity	[81]
	Chitosan nanogels	Innate immune response	Stimulation	Infectious diseases	Increased costimulatory markers (CD80, CD86) with selective cytokine profiles (IFN-γ, TNF-α, IL-1β elevation, limited IL-12)	[83]
Dendrimers		Adaptive and innate	Stimulation	Infectious diseases	Enhanced cellular uptake under acidic conditions, accelerated pro-inflammatory cytokine production (IL-1β, IL-6, TNFα)	[84]
Nanoemulsions		Innate immune response	Stimulation	Infectious diseases, cancer	Enhanced mucosal immunity with strong IgA and IgG responses, produced a broad cytokine profile (G-CSF, GM-CSF, IL-1, IL-6, IL-12)	[28,85,86,87,88,89]

**Table 2:** A representative list of nanomaterials utilized for immunomodulation with their design strategies, immune response types, mechanisms, and key findings.

Nanomaterial Type		Immune Response	Structure	Size	Purpose	Design Strategies	Mechanism	Key Findings	References
Organic nanomaterials	Polymeric NPs	Adaptive	Solid matrix	50–300	Controlled delivery of immunomodulators, antigens, or adjuvants; immune activation or suppression	Surface-targeting ligands	Biodegradable polymer degradation, enhanced DC and T-cell activation	Induction of effector memory T-cells	[90]
	PLGA nanoparticles	Adaptive	Biodegradable polymeric matrix	50–300	Sustained antigen/drug release, vaccine delivery, modulation of dendritic cells and T-cell responses	Sustained release, targeting ligands	Controlled release, enhanced uptake by APCs	Enhanced T-cell activation and macrophage activity, reduced tumor volume, increased effector and memory immune cells	[91]
Inorganic nanomaterial	Mesoporous silica NPs	Adaptive	Porous inorganic framework with tunable pore size	50–200	High-capacity loading of antigens/adjuvants, enhanced antigen presentation, cancer immunotherapy	Loading of TLR7/8 agonist	Cytokine production, endosomal trafficking	Strong DC activation, Th1/Th17 polarization, slow antigen release, high antibody titers	[92]
	Quantum dots	Innate and adaptive	Semiconductor nanocrystals	5–20	Immune cell tracking, theranostics, antigen presentation, immune tolerance induction	Core–shell fabrication, RGD modification	Apoptotic signaling, calreticulin exposure, MHC presentation	Enhanced drug delivery, induced tumor cell apoptosis, promoted DC maturation, IFN-γ/TNF-α secretion	[93]
	Calcium phosphate NPs	Adaptive and innate	Inorganic mineral-based matrix	20–100	Vaccine delivery, biocompatible antigen/adjuvant carrier, immune stimulation	~90 nm size, antigen loading, silica-coating	Costimulatory molecule upregulation, IL-6 secretion	Balanced Th1/Th2 response, potent DC activation (CD80/86), sustained T-cell immunity, enhanced mucosal IgA	[94]
	Gold nanoparticles	Innate	Solid metallic core with functionalized surface	5–100	Vaccine adjuvants, antigen delivery, immune stimulation, photothermal immunotherapy	Spherical, 40–50 nm size, surface functionalization with peptides or antibodies	Inhibition of NF-κB, MAPK pathways, TREM2 overexpression	Induced M2 macrophage polarization, enhanced cytokine production (TNF-α, IL-6), promoted autophagy and phagocytosis	[95]
	Iron oxide nanoparticles	Innate and adaptive	Magnetic inorganic core (often surface-coated)	5–100	Macrophage polarization, immune activation, imaging-guided immunotherapy	Carboxyl functionalization, dextran coating, radiotherapy combination	Complement activation, cytokine secretion (HMGB1, ATP)	Activated complement, promoted DC maturation, repolarized macrophages, enhanced tumor immune cell infiltration	[96]
Chitosan nanogels		Innate and adaptive	Crosslinked networks	20–200	Antigen and cytokine delivery, mucosal immunization, immune cell modulation	cGAS-STING pathway activation	Mitochondrial ROS production, costimulatory molecule expression	Activated DCs, increased IFN-γ and TNF-α, promoted mucosal IgA and systemic IgG responses, antibacterial properties	[90]
Dendrimers		Innate and adaptive	Branched polymers	5–50	Targeted delivery of immunomodulators, multivalent antigen presentation, immune regulation	PAMAM dendrimers, antibody conjugation, gene delivery, loading of TLR7/8 agonist	Enhanced endocytosis, pro-inflammatory cytokine production	Enhanced NK cell cytotoxicity and ADCC, delivered IL-2 pDNA activating STING pathway	[97,98]

Abbreviations: DCs—Dendritic cells, HMGB1—high-mobility group box 1 protein, TNF—tumor necrosis factor, APCs—antigen-presenting cells, MAPK—mitogen-activated protein kinase, STING—stimulator of interferon genes, RGD—arginine glycine aspartic acid, TLR—toll-like receptor, TREM2—triggering receptor expressed on myeloid cells 2, ADCC—antibody-dependent cellular cytotoxicity, and NK—natural killer.

### 2.1. Organic Nanoparticles

**Figure 1:**
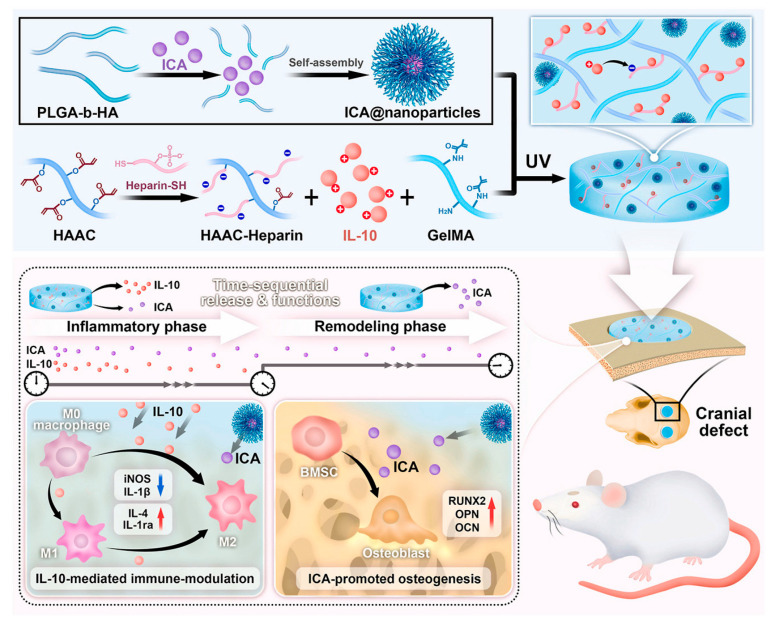
The composite hydrogel, composed of GeIMA and heparin-based acrylated hyaluronic acid (HA), incorporates PLGA-HA nanoparticles loaded with icariin (ICA). The system enables time-sequential release of IL-10 and ICA, where IL-10 is rapidly released during the early inflammatory phase to modulate the immune microenvironment, followed by sustained ICA release to promote osteogenic differentiation and bone remodeling. Black curved arrows in the IL-10-mediated immune-modulation indicate the dynamic polarization process between M0, M1, and M2 macrophage phenotypes. Red arrows represent stimulation of anti-inflammatory mediators such as IL-4 and IL-1ra. Blue arrows denote inhibitory or downregulatory effects on pro-inflammatory mediators (e.g., iNOS and IL-1β). The dashed gray arrows indicate the release pathways of bioactive factors (IL-10 and ICA) from the hydrogel system into the surrounding microenvironment. Reproduced from ref. [104] with permission from Elsevier. This work is licensed under a Creative Commons Attribution 4.0 (CC BY) International License.

Among organic nanoparticles, polymeric nanoparticles (poly lactic-co-glycolic acid, polylactic acid, chitosan) have been extensively utilized both in diagnostic and therapy applications. Poly lactic-co-glycolic acid (PLGA) NPs are widely employed as essential materials for drug delivery and immunotherapy in many FDA-approved therapeutic devices owing to their ease of synthesis, adaptability and safety. In an earlier study, colloidal gel derived from negatively charged alginate–PLGA nanoparticles and positively charged chitosan–PLGA has been utilized for suppressing experimental autoimmune encephalomyelitis (EAE) in a mouse model [99]. In this study, vaccine-like therapeutics was achieved through controlled release of the peptide Ac-PLP-BPI-NH_2_-2 from polymeric nanoparticles. The mechanism of EAE suppression using Ac-PLP-BPI-NH_2_-2-NP-treated mice was a shift in the immune response away from Th17 production, as evidenced by significantly lower production of IL-6 and IL-17. In another study, PLGA NPs containing a liver X receptor agonist (GW3965) inhibited inflammation and atherosclerosis by suppressing secretion of atherogenic factors monocyte chemoattractant protein-1 (MCP-1) and TNFα and pro-inflammatory molecules [100]. PLGA NPs are utilized not only for immune system suppression, but also stimulation of the immune system. PLGA nanoparticles containing breast tumor-derived tumor lysate antigens effectively stimulated the maturation of DCs [51]. In another study, sustained release of PLGA NPs loaded with CpG oligonucleotide enhanced myeloid-derived suppressor cells, leading to cytokine release resulting in hyperinflammation in leishmaniasis treatment [101]. Another type of a polymeric compound, polyurethane–polyurea NPs encapsulating different immunosuppressive agents, has been utilized for dendritic cell (DC) targeting [102]. Flórez-Grau et al. utilized polyurethane–polyurea NPs encapsulated with immunosuppressive lipophilic budesonide to selectively target DCs. The sustained release of immunosuppressive lipophilic budesonide resulted in downregulation of DC costimulatory receptors, which are involved in increased secretion of anti-inflammatory IL-10 by DCs and T-cell proliferation [103]. In a recent study, a multifunctional composite hydrogel/nanoparticle system was developed for cranial defect repair. Herein, hydrogel is fabricated from gelatin methacryloyl (GelMA) and heparin-based acrylated hyaluronic acid (HA), while nanoparticles are derived from PLGA-HA [104]. As shown in Figure 1, the system enables sequential delivery of an anti-inflammatory factor (IL-10) and osteogenic drug (icariin, ICA), where IL-10 is rapidly released to regulate the inflammatory phase, followed by sustained ICA release to promote osteogenesis. The results demonstrated significant enhancement of the osteogenic differentiation of bone marrow-derived mesenchymal stem cells (BMSCs) through a dual-factor-releasing nanoparticle/hydrogel hybrid system. In addition, sequential delivery of an anti-inflammatory factor (IL-10) and osteogenic drug (icariin, ICA) from the hybrid system revealed a synergistic effect for bone remodeling in a critical cranial defect rat model.

Other material classes of polymeric nanoparticles utilized mainly for immunosuppression and anti-inflammation include lipid nanoparticles (LNPs), liposomes and micelles, owing to their multifaceted drug delivery systems and improved bioavailability of therapeutics. A novel LNP, PL1 led to reactivation of the anti-tumor immune response in a number of tumor models. The anti-tumor response was achieved by effectively delivering CD134 or OX40 costimulatory receptor mRNA to tumor-infiltrating T-cells [105]. Furthermore, a bifunctional liposome was developed by phospholipid-conjugated oxaliplatin-prodrug (Oxa (IV) through self-assembly. The alkylated NLG919 (aNLG), an Indoleamine 2,3-dioxygenase 1 (IDO1) inhibitor enabled highly efficient passive tumor homing owing to long blood-circulation time. The developed liposome showed anti-tumor effect by increasing the infiltration level of CD8^+^ T-cells through decreasing immunosuppressive T-cells and inducing the secretion of cytotoxic cytokines [48]. In a recent study, cationic polymer–lipid hybridized nanovesicles increased the uptake of vaccine by DCs through stimulation of DC maturation. In addition, liposome employment acted as a benign therapeutic method for tumor immunotherapy by increasing the infiltration level of CD8^+^ T-cells [49]. Overall, polymeric nanoparticles, lipid nanoparticles, liposomes, and micelles facilitate a diverse and potent platform for immunomodulation. By enhancing antigen delivery and presentation, acting as adjuvants, targeting the delivery of immunomodulatory agents, reprogramming immune cells, and inducing immunogenic cell death, organic nanoparticles can significantly enhance the safety and efficacy of cancer immunotherapies and the treatment of infectious diseases, autoimmune disorders, and allergies.

### 2.2. Inorganic Nanoparticles

Inorganic nanoparticles offer a versatile and powerful platform for immunomodulation. By leveraging advanced design strategies such as surface functionalization, controlled release mechanisms, size and shape optimization, multifunctionality, and biocompatibility, researchers may develop sophisticated nanomaterials tailored for specific therapeutic needs. Examples of inorganic nanoparticles include gold nanoparticles (AuNPs), silica nanoparticles, iron oxide nanoparticles, carbon dots, quantum dots, etc. In an earlier study, CpG-conjugated gold nanoparticles triggered the TLR9 signaling pathway and demonstrated stimulation of macrophages for the secretion of pro-inflammatory cytokines [106]. In another study, gold nanoparticles were used as a melanoma cancer vaccine by co-delivering antigenic red fluorescent protein (RFP) along with CpG oligodeoxynucleotides (ODNs) [107]. The RFP/CpG gold nanoparticles demonstrated a strong anti-tumor effect by enhancing the proliferation of dendritic cells and T-cells through increased production of antigen-specific antibodies and interferon (IFN)-γ in melanoma models expressing RFP. Additionally, Zhou and colleagues employed optimal-sized gold nanoparticles to facilitate the migration of dendritic cells to lymph nodes, thereby promoting the activation of CD4^+^ and CD8^+^ T-cell responses [108].

Apart from gold nanoparticles, iron nanoparticles have also been utilized for immunomodulation and subsequently for disease treatment. Perica et al. developed nanoscale artificial antigen-presenting cells (aAPCs) by combining major histocompatibility complex (MHC)-immunoglobulin (Ig) dimers with signal 1 and signal 2 molecules, using dextran-coated iron nanoparticles. This nanosystem platform consists of signal 1 and signal 2, which include a foreign peptide attached to an MHC and a costimulatory molecule B7.1-Ig, designed to trigger T-cell activation and boost anti-tumor response [67]. In another study, the iron oxide nanoparticle system (FDA-approved) ferumoxytol was found to cause the polarization of macrophages toward an M1 phenotype for the treatment of mammary and lung cancers [39]. The macrophage phenotype change led to secretion of pro-inflammatory cytokines to regress tumor growth. Furthermore, a zinc-doped iron oxide nanoparticle system, coated with a phospholipid, was employed to boost the immune response by co-delivering poly(I:C) and imiquimod (R837), while concurrently activating TLR3 and TLR7 [66]. NABCD (nucleic acid-binding cyclodextrin) caused a significant suppression of the expression of innate immune activation genes, such as Mx1, Prkr, Il1b, and Tnfa, when exposed to DNA or poly I:C, according to in vitro tests conducted with bone marrow-derived macrophages (BMMs). An acute pro-inflammatory response was produced in C57BL/6 mice treated with the TLR ligand polyI:C, as evidenced by the increased production of cytokines and interferon response genes. The stimulation of the immune system indicated the secretion of pro-inflammatory cytokines and antibodies for DC maturation and activation of CTLs. Moreover, the developed immunomodulatory nanosystem demonstrated effective induction of humoral and cellular immune responses. Another nanomaterial, hollow and non-hollow mesoporous silica nanoparticles incorporating antigens and adjuvants, has also been explored for potential immunomodulation [109]. The hollow mesoporous silica nanoparticles reported improved cellular immune responses and created a superior anti-tumor effect to their non-hollow counterpart. In another study, hollow mesoporous silica nanoparticles served as a lung cancer vaccine for induction of significant tumor regression by improving the secretion of Th_1_- and Th_2_-related cytokines. In addition, CD4^+^ and CD8^+^ T-cell proliferation was enhanced using mesoporous silica nanoparticles [110].

Similarly, quantum dots (QDs) and carbon dots (CDs) have shown immunomodulatory and theranostic potential, similarly to the previously mentioned nanomaterials. Perica et al. created nano-artificial antigen-presenting cells (aAPCs) for T-cell immunotherapy by functionalizing QDs with MHC-Ig dimers and anti-CD28, leading to a notable reduction in melanoma tumor growth [111]. In another study, self-antigen (with varying surface densities)-decorated QDs were utilized for regulating immunological tolerance [75]. The results indicated more efficient induction of immune tolerance with larger QDs with low-packing-density antigens compared to their higher-antigen-density counterparts. MXenes are two-dimensional nanomaterials made up of surface terminations (Txs), carbon/nitrogen (X), and transition metals (Ms). They have become attractive options for biological applications because of their special physicochemical characteristics. These materials show improved stability, adjustable photoluminescence, and improved biocompatibility when reduced to MXene quantum dots (MQDs) [112]. MQDs provide precise control over immune responses in immunomodulation while reducing cytotoxicity. By modifying antigen-presenting cells and reducing allogeneic T-cell activation, Ta_4_C_3_Tx MQDs have shown notable in vivo effectiveness in treating transplant vasculopathy, providing a new platform for immune-targeted treatments [113]. Luo et al. further developed a potent adjuvant for cancer immunotherapy using fluorescent-labeled PEGylated CDs [30]. The combination of PEGylated carbon dots (CDs) with antigenic ovalbumin (OVA) showed improved maturation of DCs, increased antigen uptake, and enhanced secretion of tumor necrosis factor (TNF)-α, along with the expression of costimulatory molecules, leading to significant tumor regression in a melanoma model. Additionally, PEI-conjugated fluorescent CDs were created to serve as a vaccine delivery system for intranasal immunization with OVA. This system boosted the secretion of IFN-γ, as well as the IgG and IgA antibodies, and promoted splenocyte proliferation and memory T-cell development [114]. Overall, inorganic nanoparticles that integrate multiple therapies into a cohesive system hold great promise for enhancing their immunomodulatory effects. However, it is essential to address concerns related to toxicity, potential tissue damage, immunogenicity, and carcinogenesis for each nanomaterial system employed for immunomodulatory applications.

### 2.3. Nanogels

**Figure 2:**
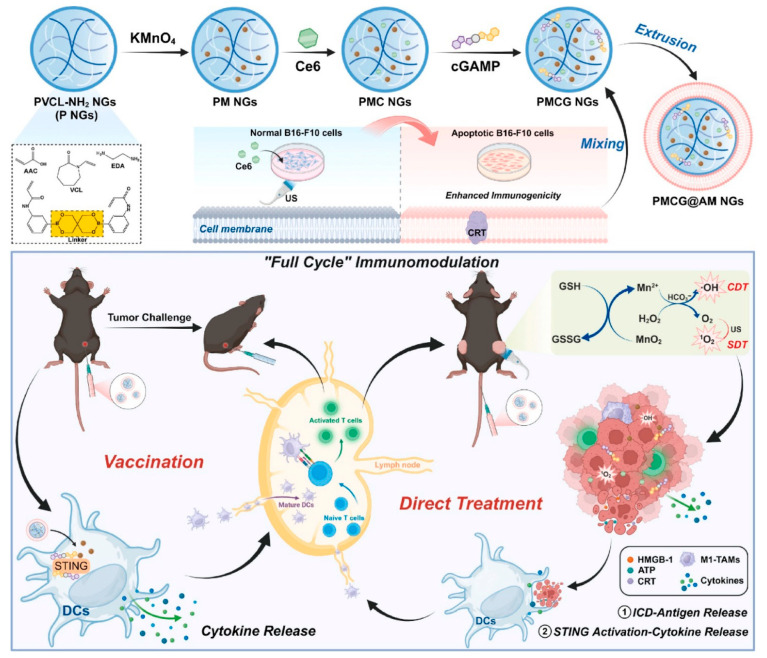
Poly(N-vinylcaprolactam) nanogels loaded with MnO2 nanoparticles in situ as PM NGs, then chlorin e6 (Ce6) was loaded and cyclic GMP-AMP (cGAMP) was coated with apoptotic cancer cell membranes (AMs) as nanovaccine to enhance recognition by antigen-presenting cells (APCs). This nanovaccine was subcutaneously injected at the left groin of healthy mice. The system activates the STING pathway to promote immune responses and T-cell activation. In the tumor microenvironment, Mn^+2^ and Ce6 are released to induce chemodynamic therapy (CDT) and sonodynamic therapy (SDT) under ultrasound irradiation, leading to immunogenic cell death (ICD), as evidenced by increased the release of high mobility group protein 1 (HMGB-1) and adenosine 5′-triphosphate (ATP), as well as calreticulin (CRT). ICD promotes the activation of Dendritic cells (DCs) which subsequently activate T-cells in the lymph nodes. This combined effects of tumor cell killing and immune activation enable full-cycle immunomodulation for the suppression of bilateral tumors. Reproduced from ref. [121] with permission from Elsevier. This work is licensed under a Creative Commons Attribution 4.0 (CC BY) International License.

Nanogels are hydrophilic vehicles that represent the second major class of nanoparticles for immunotherapy. Nanogels are characteristically soft three-dimensional networks and have shown the capability to improve circulation and biodistribution [115]. Nanogels are obtained from either natural and synthetic polymers or their combinations to develop polysaccharide nanogels, peptide nanogels, and nucleic acid nanogels, while examples of synthetic polymers include polyethylene glycol (PEG) and poly (N-isopropylacrylamide) (PNIPAM). Moreover, nanogels offer a promising platform for the precise and effective modulation of the immune system, with potential applications spanning from cancer treatment to management of autoimmune diseases. The mechanisms of immunomodulation using nanogels include antigen delivery, drug delivery, adjuvant effect, and immune cell function modulation [60,116]. In modulation of immune cell function, nanogels can be designed to modulate the function of specific immune cells (e.g., T-cells, macrophages) by delivering cytokines, siRNA, or other modulatory agents. Muraoka et al. developed a cholesteryl pullulan nanogel to mount a potent anti-tumor response by activating cytotoxic T lymphocytes (CTLs) by co-delivering peptide antigens and CpG ODN adjuvants [117]. In another study, protein nanogels made from poly (hydroxyethyl methacrylate) mixed with proteins (e.g., fibronectin or bovine serum albumin (BSA)) were utilized for delivery of ovalbumin (OVA) for generating CTLs [118]. Furthermore, a DNA-based nanogel reported sustained release of ethylenediamine-conjugated OVA and CpG ODNs for the induction of potent tumor immunity. The nanogels acted as an effective antigen delivery vehicle which significantly improved antigen presentation in dendritic cells with sustained release of OVA [119]. Apart from natural polymer-based nanogels, synthetic polymer-based nanogels have also been extensively utilized for immunomodulation and disease treatment. In 2016, researchers employed a PEG-like polymer along with a hydrophobic fluoropolymer nanogel, loaded with imidazoquinoline (a TLR7/8 agonist), to stimulate T-cell responses to a tuberculosis antigen. This approach demonstrated improved adjuvanticity and reduced systemic toxicity [120]. In another study, a nanogel system made from hyaluronic acid (HA) linked with polyethyleneimine (PEI) enhanced the humoral immune response by co-delivering the antigenic ovalbumin (OVA) and cyclic guanosine monophosphate adenosine monophosphate [54]. In this study, methotrexate (MTX)-loaded nanogels composed of gold nanoparticles and diselenide-crosslinked poly(N-vinylcaprolactam) (PVCL) were used for immunomodulation-mediated enhanced chemotherapy [60]. The results showed the induction of macrophage repolarization from a protumor M2-like phenotype to an anti-tumor M1-like phenotype in vitro with stimuli-responsive release of Au NPs and MTX. As a result, there was prevention of DNA replication with the promotion of cancer cell apoptosis. Furthermore, remodeling of tumor-associated macrophages to the M1-like phenotype was indicated in a subcutaneous mouse melanoma model using developed nanogels in in vivo studies. Moreover, increased effector T lymphocyte recruitment and a reduction in immunosuppressive regulatory T-cells was attained through remodeling of TAMs, which suggests synergistic enhancement of anti-tumor efficacy using combination therapy. In a recent study, an advanced therapeutic nanovaccine formulation was developed using on poly (N-vinylcaprolactam) nanogels (NGs) loaded with manganese dioxide (MnO_2_), the sonosensitizer chlorin e6 (Ce6), and the immune adjuvant cyclic GMP-AMP (cGAMP), along with coating of apoptotic cancer cell membranes (AM)-(PMCG-AM nanogels) to tackle bilateral tumor growth through full-cycle immunomodulation [121]. As illustrated in Figure 2, the biomimetic NG system enables a full-cycle immunomodulation strategy. The AM coating enhances recognition and uptake by antigen-presenting cells (APCs), while Mn^+2^ and cGAMP activate the STING pathway to promote immune activation and T-cell priming. Simultaneously, within the tumor microenvironment, the NGs release Mn^+2^ and Ce6, enabling chemodynamic and sonodynamic therapy under ultrasound irradiation, which induces immunogenic cell death (ICD). The combination of local tumor destruction and systemic immune activation results in a synergistic effect that suppresses tumor growth. The presence of these therapeutic agents allows the NGs to activate T-cells to prevent tumor growth through STING activation and antigen presentation.

### 2.4. Nanocapsules

Nanocapsules, similarly to nanogels, hold significant promise for immunomodulation due to their distinct structural and functional characteristics. The nanocapsules are often designed with a core–shell structure, where the core houses the therapeutic agent, while the protective shell can be customized for targeted delivery [23]. Core–shell nanocapsules, made from biocompatible materials like lipids, PLGA, or proteins, enable controlled, sustained release and targeted delivery of therapeutic agents or antigens [122]. They increase the precision of immunomodulation while reducing toxicity and off-target effects by improving antigen presentation, co-delivering adjuvants, and modifying immune cells like APCs.

**Figure 3:**
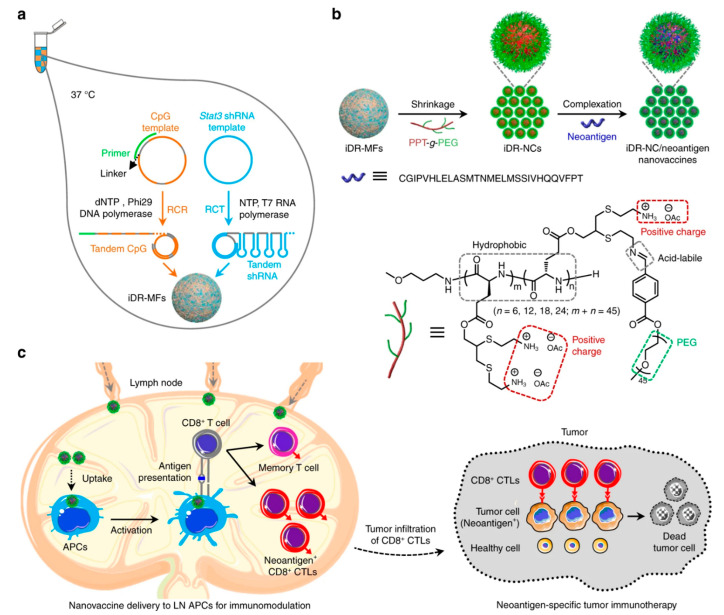
Schematics showing intertwining DNA-RNA nanocapsules conjugated with neoantigens (iDR-NC/neoantigen) as nanovaccines for synergistic tumor immunotherapy. (**a**) Generation of tandem CpG and Stat3 shRNA via concurrent rolling-circle replication (RCR) and rolling-circle transcription (RCT) in the same solution to construct self-assembly into intertwining DNA-RNA microflowers (MFs), (**b**) shrinkage of these MFs to form iDR-NCs using PEG-grafted polypeptide (PPT-g-PEG) followed by loading of a tumor-specific neoantigen via hydrophobic interactions, (**c**) delivery of iDR-NCs/neoantigen complexes into APCs in draining lymph nodes (LNs) and inhibition of tumor progression in immunocompetent mice by eliciting potent and durable neoantigen-specific T-cell responses. Reproduced from ref. [131] with permission from Nature Publishing Group. This work is licensed under a Creative Commons Attribution 4.0 (CC BY) International License.

Nanocapsules are one of the earliest and most widely utilized nanomaterials in clinical research, offering promising potential for clinical use due to their well-defined properties, controlled release profiles, and scalability in production [123]. A primary form of nanocapsules studied for immunomodulatory applications includes nanoparticles resembling liposomes. Their supramolecular structure supports weaker intermolecular forces, which facilitates a higher loading capacity than that of traditional liposomes [124,125]. Furthermore, similar to liposomes, a lipid-based nanocage ISCOMATRIX (made from phospholipids, saponin, and cholesterol) with potent immunomodulatory effects has been utilized for adjuvant delivery for therapeutic and prophylactic vaccines against infectious diseases [126,127]. In 2015, Jewell created polyelectrolyte multilayer (PEM) nanocapsules to co-deliver positively charged OVA peptide antigens and negatively charged poly(I:C) adjuvants, aiming to inhibit tumor growth by activating T-cells in a melanoma model [128]. In another study, PEM nanocapsules loaded with myelin (a self-antigen) and CpG oligodeoxynucleotides (ODNs), TLR antagonists, promoted immune tolerance [129,130]. The engineered nanocapsules promote immune tolerance in autoimmune conditions, like multiple sclerosis, by reducing the activation of dendritic cells and steering myelin-specific T-cells toward tolerogenic states. Furthermore, studies have investigated intertwining DNA-RNA nanocapsules (iDR-NCs) with tumor-specific neoantigens as nanovaccines for synergistic cancer immunotherapy [131]. iDR-NCs are generated through self-assembly of DNA and RNA microflowers via concurrent rolling-circle replication (RCR) and rolling-circle transcription (RCT), followed by shrinkage using PEG-grafted cationic polypeptides and subsequent loading of neoantigens. As illustrated in Figure 3, the resulting iDR-NC/neoantigen complexes enable efficient co-delivery of CPG and Stat3 shRNA adjuvants together with tumor-specific antigens into antigen-presenting cells (APCs) in lymph nodes. This combined delivery promotes APC activation and sustained antigen presentation, leading to enhanced neoantigen-specific immune responses. Consequently, the developed iDR-NCs elicited 8-fold more frequent peripheral CD8^+^ T-cells and significantly inhibited the progression of colorectal tumors. Kulkarni and co-workers developed liposome-like nanoparticles, which help in repolarising TAMs from an M2 phenotype to an M1 phenotype for inhibition of colony-stimulating factor 1 receptor (CSF-1R) in tumors [124]. In another study, the Jewell group demonstrated that PEM nanocapsules loaded with antigens and TLR agonists significantly accelerated dendritic cell uptake, processing, and activation to support immunomodulation. [132]. Overall, nanocapsule systems are emerging immunomodulator nanomaterials and addressing the relationship between the longevity of immune protection or activity and the release of encapsulated cargo needs special attention.

### 2.5. Nanoemulsions

Nanoemulsions are emulsions with droplet sizes that are generally between 20 and 200 nanometers. Their small scale provides unique characteristics, including a high surface area, increased solubility for hydrophobic drugs, and enhanced bioavailability. These characteristics make nanoemulsions a promising platform for immunomodulation, which involves modifying the immune response to achieve a therapeutic effect. Nanoemulsions represent a versatile and promising platform for immunomodulation, offering enhanced delivery, controlled release, and the potential for combination therapies [24]. Nanoemulsions enhance the delivery, stability, and bioavailability of immunomodulatory agents, enabling targeted, controlled, and sustained release for treating cancer, autoimmune, and infectious diseases. They are extremely adaptable in immunotherapy due to their capacity to solubilize hydrophobic drugs, co-deliver numerous drugs, act as adjuvants in vaccines, and either stimulate or inhibit immune responses.

Zeng et al. developed functionalized nanoemulsions encapsulating tumor-related antigens to target Clec9A (six neoepitopes) for cancer immunotherapy. The developed nanoemulsion significantly suppressed the growth of B16-F10 melanoma in a CD4^+^ T-cell-dependent manner [133]. In another study, a nanoemulsion (NE)-based immunotherapeutic platform was developed for modulation of tumor-induced suppression as well as induction of T-cell proliferation via effective cell-mediated immune response. In this study, a nanoemulsion loaded with TLR7/8 agonists served as a vaccine adjuvant and significantly induced the activation of innate immune cells, thus promoting polarization of M2 macrophages and increasing the infiltration level of lymphocytes [86]. As a result, significant tumor growth inhibition and improved survival outcomes were observed in a mouse tumor model [86]. Furthermore, a composite nanoemulsion encapsulating a protein vaccine by magnetic ultrasound technology has been employed for therapy. The nanoemulsion–protein vaccine composite (M1M3MnH) demonstrated improved outcomes in combination therapy in cancer treatment by providing a stronger anti-tumor immune response [87]. In a recent study, researchers developed a cancer vaccine adjuvanted with a TLR7/8 agonist (R848)-loaded nanoemulsion to reprogram suppressive immune cells to exert an anti-tumor immune response [89]. The mechanism of anti-tumor immune response includes T-cell activation and inhibition of T-cell depletion to prevent recurrence and tumor metastasis in a lung cancer model. Furthermore, an aluminum–phthalocyanine-mediated nanoemulsion demonstrated the effective inhibition of tumor growth and lung metastasis in a murine model [88,134]. Nevertheless, nanoemulsion-based immunomodulation has demonstrated better clinical outcomes in disease treatment, especially cancer. However, scaling up, they still possess challenges of safety, toxicity and regulatory hurdles in their clinical use.

### 2.6. Dendrimers

Dendrimers are highly branched, nanoscale, tree-like structures with a well-defined, uniform, and monodisperse architecture. Due to their unique properties, including functionality, multivalency, and ability for precise control over size and shape, dendrimers are emerging as powerful tools for immunomodulation. There are various ways dendrimers can be utilized for immunomodulatory purposes viz., enhanced delivery of immunomodulatory agents, targeted delivery, adjuvant effects, controlled release, modulation of immune functions, and combination therapy [55]. Dendrimers improve stability, bioavailability, and cellular absorption by facilitating the targeted, regulated delivery of drugs by core encapsulation and surface functionalization. They are useful in the treatment of cancer, infections, and autoimmune and inflammatory diseases because of their ability to co-deliver various drugs, respond to stimuli, function as vaccine adjuvants, and target certain immune cells. In 2013, an HIV-peptide-loaded dendrimer conjugated with maltose-decorated poly (propylene imine) was reported to deliver antigenic peptides to dendritic cells without inducing any cytotoxic effects. The delivery of peptides to dendritic cells promoted cytokine secretion and CTL activation in in vitro studies [84]. Dendrimers overexpress folate receptors and specifically target macrophages under inflammatory settings through the delivery of drugs for rheumatoid arthritis treatment. In an earlier study, G5 polyamidoamine (PAMAM) dendrimer–methotrexate (MTX) conjugates bound to folic acid efficiently transported the drug to the inflammasome to specifically target macrophages in a concentration- and temperature-dependent manner [135,136]. The regulation of monocyte and macrophage activity attributes an anti-inflammatory role to dendrimers. Furthermore, azabisphosphonate (ABP) dendrimers represent the most exploited nanosystem for the treatment of musculoskeletal diseases by treating neuro-inflammation through the promotion of IL-10-producing CD4^+^ T lymphocytes [137]. Beyond traditional dendrimers, PEGylated lipid-coated ionizable dendrimers, referred to as modified dendrimer nanoparticles (MDNPs), have been developed to deliver antigenic mRNA for treating infectious diseases such as Ebola, H1N1 influenza, and *Toxoplasma gondii* [138]. In 2017, Whittum-Hudson and colleagues introduced a peptide-conjugated PAMAM dendrimer targeting the sexually transmitted bacterium *Chlamydia trachomatis* [139]. The dendrimer backbone combined with antigenic peptides that mimic chlamydial glycolipid antigens significantly enhanced peptide immunogenicity and triggered a strong humoral immune response by providing controlled release of the therapeutic cargo. Recently, several immunotherapeutic strategies have been utilized as new generation of cancer therapy using dendrimer/branched polyethyleneimine (PEI)-based cancer vaccines. The developed vaccines induced specific anti-tumor immune responses by combining tumor-associated antigens and immune adjuvants [55]. Dendrimers offer a highly versatile platform for immunomodulation due to their multivalency, precise structure, and ease of modification, showing promise in cancer, autoimmune, and infectious disease therapy. Despite concerns about biodistribution and safety, tailored designs enhance bioavailability and immune activation, warranting further research for clinical translation.

### 2.7. Extracellular Vesicles

Extracellular vesicles (EVs) are lipid-based nanomaterials that are spontaneously released by cells. They include biomolecules like proteins, metabolites, DNA, and RNA that are indicative of the physiological condition of their parent cells. They are effective therapeutic intervention tools because of their functions in immune regulation and intercellular communication. By changing the behavior of recipient cells, EVs produced from sources such as cancer cells, infected cells, or mesenchymal stromal cells (MSCs) might affect immunological responses [140]. Notably, in immunosuppressed mice, EV-like nanoparticles derived from marine macroalgae (*Sargassum fusiforme*) have been demonstrated to improve dendritic cell maturation and T-cell activation, thereby regaining immunological function [141]. Similar to this, MSC-derived EVs have potent immunosuppressive qualities by preventing lymphocyte proliferation, especially when preconditioned with cytokines or genetically modified [142]. Depending on their origin and engineering, EVs can either activate or suppress the immune system, as these studies highlight. This makes them extremely adaptable for immunomodulatory treatments.

## 3. Mechanisms of Immunomodulation Using Nanomaterials

Nanomaterials leverage their role in modulating the immune system through immunostimulation or immunosuppression, based on therapeutic needs. They affect responses including inflammation, tolerance, and cell activation by interacting with both innate and adaptive immune cells [1]. The delivery of antigens, the adjuvant effects, and the regulated release of immunomodulatory substances are important factors [1].

**Figure 4:**
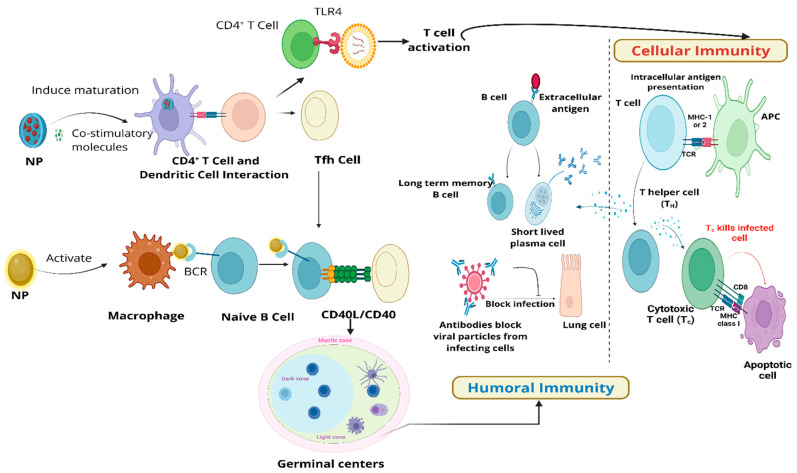
Nanoparticle (NP)-mediated activation of adaptive immunity through coordinated humoral and cellular immune responses. In the humoral arm (left), NPs drive dendritic cell (DC) maturation by upregulating co-stimulatory molecules (CD80, CD86) and engaging Toll-like receptor 4 (TLR4) signaling during DC–CD4^+^ T cell interactions. Primed CD4^+^ T cells differentiate into T follicular helper (Tfh) cells, which deliver cognate CD40 ligand (CD40L)–CD40 help to naive B cells; concurrently, NP-activated macrophages facilitate antigen delivery to B cells via B cell receptor (BCR) signaling. Activated B cells enter germinal centers—comprising dark, light, and mantle zones—where affinity maturation generates short-lived plasma cells and long-lived memory B cells. Plasma cells secrete antigen-specific antibodies that neutralize extracellular pathogens and block infection of target tissues. In the cellular arm (right), antigen-presenting cells (APCs) present intracellular antigens on major histocompatibility complex class I (MHC-I) or class II (MHC-II) molecules, engaging T cell receptors (TCR) to generate T helper (T_H_) and cytotoxic T lymphocyte (CTL, CD8^+^) populations; CTLs recognize MHC-I–peptide complexes via TCR and CD8 co-receptor and eliminate infected cells through apoptosis. Together, these two arms constitute complementary nanomaterial-orchestrated adaptive immune responses capable of providing both humoral protection and cell-mediated cytotoxicity. Created in BioRender. Kapre, S. (2026) https://BioRender.com/efsijdp (accessed on 28 February 2026).

The mechanisms induced by nanomaterials to modulate the immune system include stress mechanisms and the activation of cell signaling pathways (Figure 4). The stress mechanism involves oxidative stress, which represents the main downstream event of inflammation. Oxidative stress caused by nanomaterials leads to excessive production of reactive oxygen species (ROS) compared to their bulk form, owing to their higher intrinsic reactivity and larger surface area [143]. Nanomaterial-induced oxidative damage demonstrated enhancement in immune function or inflammatory response in various in vitro and in vivo studies [69,77,144,145,146]. Nanoparticle-induced ROS production plays an important role in inflammatory diseases via a cascade of pathophysiological consequences related to activation of associated cell signaling pathways and inflammation [146,147]. Oxidative stress can be influenced by several parameters, such as the nanomaterial’s dissolution rate, size and surface coating, and thus the immunomodulatory effects of nanomaterials.

An important response of the immune system includes nanomaterial-induced inflammation, as evidenced by the production of cytokines or chemokines. Moreover, induction of inflammation and cytokine production characterize the activated immune system. The release of pro-inflammatory mediators, including IL-2, IL-8, IL-6, and TNF-α, occurs through the activation of molecular pathways such as mitogen-activated protein kinase (MAPK), nuclear Factor-kB (NF-kB), and phosphoinositide 3-kinase (PI3-K). Studies conducted both in vitro and in vivo with various metal oxide nanoparticles have shown that this cytokine production can lead to toxicity [1,38]. MAPKs are oxidant-dependent signaling molecules that play a vital role in NP-induced oxidative-stress-generated inflammation via the activation of pro-inflammatory cytokine expression by stimulation of the NF-kB pathway. In addition to MAPKs, the activation of the inflammasome induces ROS-induced inflammation. In an earlier study, AgNPs in THP-1 cells demonstrated increased gene expression of IL-1, IL-6, IL-1β and TNF-α leading to activation of the inflammasome [38,148]. There are two mechanisms, namely the regulation of the immune response through modulation of immune cells and the clearance of inflammatory markers such as ROS and cytokines, which are utilized for the alleviation of nanomaterial-induced inflammation. Enhancing or suppressing inflammation includes pro-inflammatory activation and anti-inflammatory effects. In cases where enhanced immune activation is needed (e.g., cancer or infections), nanomaterials can be used to deliver pro-inflammatory agents that boost the immune response. Conversely, nanomaterials can deliver anti-inflammatory agents to suppress excessive inflammation, which is beneficial in conditions such as chronic inflammatory diseases or transplant rejection.

As discussed above, nanomaterial-exposure-generated stress leads to the initiation of several cell signaling pathways for immune system modulation. The signaling molecules and pathways include TLRs, NF-kB, and MAPKs, which are important for different types of nanomaterials. The activation of these molecular pathways leads to the activation of a cascade of processes, such as increased macrophage phagocytosis, upregulated cytokine production, and enhanced antigen presentation via the upregulation of MHC [1,149]. In one study, graphene oxide activated the TLR4 signaling pathway and decreased macrophage viability as well as inducing necrosis [150]. TLR is an innate immune system receptor and its activation triggers innate immunity, which leads to strong adaptive immunity [151]. In an earlier report, quantum dots activated the primary response gene 88, related to myeloid differentiation (MyD88, an adapter protein), for TLR-dependent-NF-*κ*B activation in macrophages [76]. Another key regulator of immune response, the NF-*κ*B pathway, mediates the synthesis of pro-inflammatory cytokines such as IL-1*β*, IL-6, IL-8, and TNF-*α* [152,153,154,155]. The interaction of nanomaterials with TLRs leads to an immunosuppressive effect, as shown by AuNPs [156]. TLR9-regulated immunosuppression inhibited the production of pro-inflammatory cytokines when using small AuNPs (4 nm) [157]. In another study, Ag nanoparticles inhibited TLR2 in a dose-dependent manner by reducing the release of pro-inflammatory cytokines (IL-1β, TNF-a and IL-6) in THP-1 cells [78].

The initial inflammatory response, which activates the inflammatory cascade, serves as a wake-up signal for innate immune receptors such as TLRs and PLRs. In 2017, Gomez et al. demonstrated that silica nanoparticles could stimulate an antiviral innate immune response by amplifying this cascade mechanism. Key players in triggering downstream inflammatory pathways are the pro-inflammatory cytokines IL-1β and IL-18 [80]. The maturation and activation of these cytokines involves a complex inflammasome pathway. The NLRP3 (NOD-, LRR- and pyrin domain-containing protein 3)-mediated inflammasome complex pathway represents the best-known pathway for triggering a cascade mechanism. The cascade leads to the induction of pro-inflammatory cytokine secretion (For ex., IL-1β and IL-18) through formation of NLRP3/pro-caspase-1 complex.

Nanomaterials can be designed with surface modifications, such as by attaching targeting ligands (like antibodies or peptides), to deliver antigens specifically to antigen-presenting cells (APCs) like dendritic cells (DCs) and macrophages [1,38]. This targeting promotes endocytosis, where the nanomaterial–antigen complex is internalized by the APCs. Macrophage polarization plays a vital role in inflammatory diseases, as macrophages can adopt either an inflammatory (M1) or wound-healing (M2) phenotype based on the inflammatory environment. M1 macrophages release pro-inflammatory cytokines and chemokines, intensifying inflammation, while M2 macrophages produce anti-inflammatory cytokines, helping to resolve inflammation [158]. To support this balance, various nanomaterial formulations have been developed to promote M2 polarization and limit M1 activation [29]. Additionally, nanomaterials can influence the maturation and activity of APCs like DCs, which connect the innate and adaptive immune systems due to their diverse antigen-presenting roles [63,64]. DCs are a heterogeneous class of antigen-presenting cells and the point of contact between the innate and the adaptive immune system. The maintenance of a delicate balance between immunity and tolerance is mediated by DCs. Cifuentes-Rius and co-workers utilized nanomaterials to target DCs to induce immune tolerance for tolerogenic immunotherapies through DC reprogramming [33]. Induction of immune tolerance is utilized for the treatment of autoimmune diseases and allergy. In the case of autoimmune diseases, nanomaterials can be used to deliver autoantigens in a way that promotes immune tolerance, rather than activation through antigen presentation in a non-inflammatory context. Antigen presentation in such a manner leads to the generation of regulatory T-cells that suppress the autoimmune response. Nanomaterials can be used to deliver allergens in a controlled manner to induce tolerance and reduce allergic reactions by retraining the immune system to recognize the allergen as harmless.

Modulation of immune cell behavior targeting and reprogramming of immune cells leads to either induction or suppression of immune responses. Interaction of nanomaterials with immune cells can stimulate the production of cytokines, which are signaling molecules that regulate and amplify the immune response through the activation and proliferation of T-cells and B-cells [1,38]. Some nanomaterials facilitate cross-presentation, where extracellular antigens are processed and presented on MHC class I molecules, which is crucial for activating cytotoxic T-cells to mount a robust immune response against tumors or intracellular pathogens. Nanomaterials can be functionalized to target specific subsets of immune cells, such as regulatory T-cells (Tregs) for immunosuppression or cytotoxic T-cells for immunostimulation, to modulate the immune response more precisely. Earlier reports demonstrated that nanomaterials can modulate immune cell homeostasis by shifting the Th1/Th2 balance [74,159,160]. In other studies, accentuation of Th cell immunity was achieved through inappropriate maturation/activation DCs, including Th2 (IL-4↑, IL-5↑, and IL-13↑) and Th17 (IL-17A↑, IL-23↑) using single-walled carbon nanotubes (SWCNTs) [63,64]. Meanwhile, these pathways also include the facilitation of B-cell receptor aggregation via the spatial organization of antigens on the particle surface and repetitive antigen display.

Nanomaterials can serve as adjuvants by themselves by activating pattern-recognition receptors (PRRs) such as TLRs present on the surface of immune cells, leading to an enhanced immune response. The mechanisms which explain the immunostimulatory effect of nanomaterials as adjuvants include dendritic cell membrane perturbation, TLR-dependent signal transduction, activation of the NLR family pyrin domain-containing-3 (NLRP3) inflammasome, the depot effect for maintaining the stability of antigens, autophagic regulation, and antigen delivery to draining lymph nodes [161]. Nanomaterials can also be engineered to deliver immunomodulatory agents—such as cytokines, small-molecule drugs, or siRNA—in a controlled, sustained manner. This allows for a prolonged immune response or modulation, decreasing the frequency of dosing required. Certain nanomaterials are designed to release their therapeutic cargo in response to specific triggers, like pH shifts, temperature changes, or enzyme activity, at the target location. This targeted release improves effectiveness while reducing potential side effects throughout the body. Overall, nanomaterials offer versatile platforms for immunomodulation by enhancing or suppressing the immune response through targeted delivery, controlled release, and precise interaction with immune cells. The ability to functionalize nanomaterials for specific immune pathways provides significant potential for developing advanced therapies for cancer, autoimmune diseases, infectious diseases, and more. However, careful consideration of safety, targeting, and regulatory aspects is crucial for translating these promising strategies into clinical applications.

Significantly, the biodistribution, tissue accumulation, and cellular uptake of nanomaterials in vivo have a significant impact on the immunomodulatory pathways mentioned above, which determine the strength and localization of immune responses. Complementary quantitative and imaging-based methods, such as fluorescence and bioluminescence imaging, positron emission tomography (PET), single-photon emission computed tomography (SPECT), magnetic resonance imaging (MRI), and elemental analysis by inductively coupled plasma mass spectrometry (ICP-MS), can be used to study tissue distribution, allowing spatial and temporal tracking of nanomaterials across organs [162]. By combining whole-organ biodistribution studies with cell-level analyses, such as flow cytometry, imaging flow cytometry, and mass cytometry following tissue dissociation, it is possible to quantify the accumulation of nanomaterials in particular organs and immune cell populations. This allows for the evaluation of uptake by macrophages, dendritic cells, neutrophils, and lymphocytes [163].

At the cellular level, nanomaterial–immune cell interactions involve opsonization, receptor-mediated recognition, endocytosis or phagocytosis, and modulation of immune signaling pathways, which can result in immune activation, tolerance, or suppression. These processes can be studied in vivo using intravital microscopy, multimodal imaging, and reporter animal models [164,165]. Collectively, integrating biodistribution analysis with immune cell-specific in vivo approaches is essential for linking nanomaterial localization to immune interactions and therapeutic outcomes.

## 4. Interactions of Nanomaterials with Different Immune System Components

**Figure 5:**
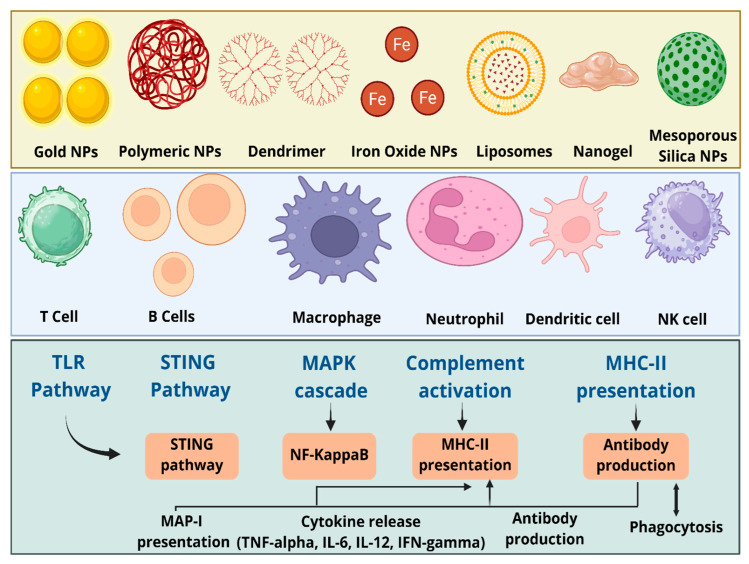
Illustration of nanomaterial types, immune cell targets, and intracellular signaling pathways implicated in nanoparticle (NP)-mediated immunomodulation. The top panel illustrates seven classes of immunomodulatory nanomaterials—gold NPs, polymeric NPs, dendrimers, iron oxide NPs, liposomes, nanogels, and mesoporous silica NPs—each offering distinct physicochemical properties that govern their immunological interactions. The middle panel depicts the primary immune cell populations engaged by these nanomaterials, encompassing both innate effectors (macrophages, neutrophils, dendritic cells, and natural killer cells) and adaptive immune cells (T and B cells). The bottom panel delineates the key intracellular signaling cascades activated upon nanomaterial–immune cell interaction: Toll-like receptor (TLR) and stimulator of interferon genes (STING) pathway activation, mitogen-activated protein kinase (MAPK) cascade–driven NF-κB transcription, complement system activation leading to MHC class II (MHC-II) antigen presentation, and downstream MHC-II–mediated antibody production and phagocytosis. These converging pathways collectively drive pro-inflammatory cytokine release (TNF-α, IL-6, IL-12, IFN-γ), antigen presentation, and adaptive antibody responses, highlighting the capacity of engineered nanomaterials to precisely modulate both innate and adaptive immunity. IL-6—interleukin 6, IFN—interferon, TNF—tumor necrosis factor, MHC—major histocompatibility complex, NK—natural killer, TLR—Created in BioRender. Thalla, M. (2026) https://BioRender.com/f3irfqg (accessed on 28 February 2026).

The immune system comprises various organs, cells, and molecules that can be altered by interactions with certain nanomaterial formulations. Targeting both innate (such as macrophages, monocytes, neutrophils, and NK cells) and adaptive (such as T- and B-cells) immune components allows for precise immunomodulatory effects when nanomaterials are engineered appropriately (Figure 5). Through their effects on immune cell activity and molecular pathways, these interactions provide the theoretical basis for therapeutics based on nanomaterials. Monocytes and macrophages are among the first cells to respond to nanomaterials. They ingest them through phagocytosis and release cytokines that can cause inflammation, such as IL-6 and TNF-α. Adaptive immunity can be initiated by DCs, which can absorb nanomaterials, mature, and present antigens to T-cells. When exposed to nanomaterials, neutrophils can phagocytose them, produce ROS and enzymes, or form neutrophil extracellular traps (NETs), which can aid in tissue injury or pathogen removal.

Additionally, nanomaterials can increase the cytotoxicity of NK cells against malignant or diseased cells. They can deliver antigens to T-cells via DCs, which will cause T-cell proliferation and the production of cytokines like IL-2 and IFN-γ. Furthermore, nanomaterials help B-cells recognize antigens, which results in the generation of antibodies and the development of memory B-cells for sustained immunity. Designing safe, efficient nanomedicines to treat immune-related, infectious, and inflammatory diseases requires an understanding of these molecular and cellular interactions (Table 3).

### 4.1. Interactions Between Nanomaterials and Macrophages

**Figure 6:**
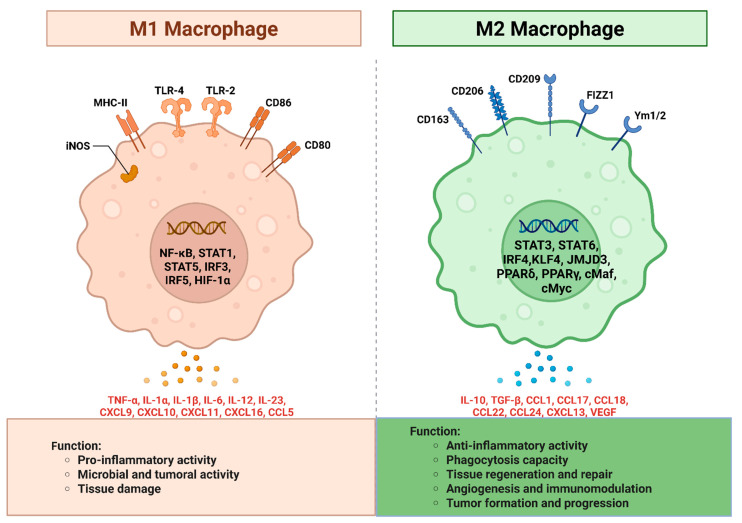
Schematic illustration of M1-like and M2-like tumor-associated macrophages (TAMs). M1-like TAMs exhibit pro-inflammatory functions and are associated with anti-tumor activity through the secretion of inflammatory cytokines and the activation of immune responses. In contrast, M2-like TAMs display anti-inflammatory properties, promoting tumor progression, immune suppression and tissue remodeling. Distinct surface markers and secreted factors characterize each phenotype, contributing to their opposing roles in regulating tumor progression and the TME. These opposing phenotypes highlight the importance of macrophage polarization as a therapeutic target for modulating the tumor microenvironment. Created in BioRender. Thalla, M. (2026) https://BioRender.com/fhtd6l3 (accessed on 28 February 2026).

Macrophages play a crucial role in the immune system by identifying, engulfing, and eliminating pathogens and foreign substances. These immune cells are highly diverse and are essential for maintaining homeostasis, supporting development, aiding tissue repair, and contributing to the immunity of an organism [173,174]. Additionally, macrophages are vital components of the innate immune system. Based on their activation and functional characteristics, macrophages can be categorized into two types: classically activated (M1) and alternatively activated (M2) (Figure 6) [175]. M1 macrophages are associated with pro-inflammatory responses and release various cytokines such as interleukin (IL)-1β, IL-6, IL-12, IL-23, and TNF-α, which are important for antigen presentation [176]. M1 macrophages are typically polarized by lipopolysaccharide (LPS), either on its own or in combination with various cytokines produced by activated T-helper 1 (Th1) cells, such as granulocyte–macrophage colony-stimulating factor (GM-CSF) and interferon-γ (IFN-γ). In contrast, M2 macrophages are characterized by their anti-inflammatory and immunoregulatory functions, as they produce anti-inflammatory cytokines like IL-10 and transforming growth factor-β (TGF-β) [175,176,177]. M2 macrophages are activated by cytokines such as IL-4 and IL-13, which are secreted by T-helper 2 (Th2) cells. The balance between M1 and M2 macrophages plays a crucial role in maintaining the homeostasis of pro-inflammatory and anti-inflammatory responses in the body. Although the underlying mechanisms are not completely understood, macrophage activation plays a critical role in physiological processes such as inflammatory disease progression, infections, and cancer [174,176].

Understanding how nanomaterials interact with macrophages helps in designing effective drug delivery systems, diagnostic tools, and therapeutic agents. Nanomaterials should be decorated with various surface ligands such as small molecules (e.g., mannose, legumain), monoclonal antibodies, peptides, and oligomers to simultaneously target macrophages and modulate their functions [178]. The interactions of nanomaterials and macrophages result in the modulation of polarization, macrophage mobilization, recruitment processes, etc. As described earlier, macrophage polarization plays a crucial role in the pathogenesis of multiple inflammatory diseases. Macrophages can polarize to M1 or M2 phenotypes based on stimuli received from the inflammatory microenvironment. M1-type macrophage cells secrete various pro-inflammatory cytokines and chemokines to attract other cell types at the inflamed site, which makes the inflammation conditions even worse. On the other hand, M2 macrophage cells act oppositely and relieve inflammatory conditions by releasing anti-inflammatory cytokines [158]. In this context, various nanomaterial formulations, such as metal nanoparticles and polymeric nanoparticles, are being used to reduce the population of M1 macrophages by promoting polarization toward the M2 subtype [29,179]. A 2017 study found that mesoporous silica nanoparticles with extra-large pores (XL-MSNs) could alleviate inflammatory conditions by delivering the M2-polarizing cytokine IL-4 to macrophages, thereby confirming M2 polarization in vivo [71]. Beyond silica nanoparticles, metallic nanoparticles, particularly AuNPs, have also been shown to affect macrophage polarization and demonstrate anti-inflammatory effects in a mouse model [180]. Previous research indicated that AuNPs served as an adjuvant to antibiotics in treating bacterial infections in a mouse cecal ligation puncture model, leading to increased M2 macrophages (CD206^+ve^ in F4/80^+ve^ cells) and decreased M1 macrophages (CD86^+ve^ in F4/80^+ve^ cells) in the spleens of sepsis-affected mice [181]. Furthermore, both natural and synthetic immunomodulatory nanoparticles are being explored to repolarize macrophages by delivering plasmid DNA or microRNA. Studies have shown that hyaluronic acid-poly(ethyleneimine) nanoparticles, loaded with IL-4/IL-10 and plasmid DNA or microRNA-223, can effectively reprogram macrophages from the M1 to the M2 subtype. The findings indicated macrophage polarization modulation, evidenced by increased CD206 expression, elevated arginine levels, reduced iNOS levels, and caused downregulation of the M1 marker CD86 [182,183]. Additionally, the mobilization and recruitment of macrophages can also be influenced by various nanomaterials. Chemokines such as chemokine C–C motif ligand 7 (CCL7) and receptors like angiotensin II (Ang II) are involved in mobilizing and recruiting macrophages during chronic inflammation [184,185]. Once mobilized, macrophages accumulate in inflamed regions where nanoparticles can exert anti-inflammatory effects [186,187]. In 2020, researchers reported that CCR2 short hairpin RNA (shRNA)-loaded EGFP-EGF1-conjugated poly(lactic-co-glycolic acid) (PLGA) nanoparticles could modulate macrophage-related inflammation in atherosclerosis by selectively knocking down CCR2 expression [188]. Apart from these, nanomaterials have also been employed in cancer immunotherapy to target macrophages. Tumor-associated macrophages (TAMs) are the most abundant among the immune cells and are recruited to the tumor site throughout tumor progression [189]. TAMs are abundant within the tumor immune microenvironment (TIME) and significantly contribute to tumor initiation, progression, and metastasis. Nanomaterials can modulate the population of tumor-promoting M2-type TAMs to counteract the immunosuppressive TIME by eliminating TAMs, preventing the infiltration of TAMs, enhancing TAM phagocytosis, and modulating the polarization of TAMs [44,190]. The modulation of tumor-promoting M2-type TAMs promotes tumor-suppressing M1-type TAMs and enhances TAM phagocytic activity through polarization. A multifaceted approach modulating TAMs has been explored for inhibiting the development and metastasis of digestive system tumors [191]. Overall, understanding these interactions helps in designing nanomaterials for biomedical applications, ensuring they are effective while minimizing potential adverse effects on the immune system.

### 4.2. Interactions Between Nanomaterials and Neutrophils

Neutrophils, a type of white blood cell, are another key component of the innate immune system, rapidly responding to infection sites and engulfing pathogens through a process called phagocytosis [15,192]. The frontline role in host defense of neutrophils suggests its immune-modulating capabilities. Though neutrophils are the most abundant leukocyte in the blood, nanomaterial interactions with neutrophils for immunomodulation and the treatment of diseases has garnered less attention as compared to macrophages. The interactions between nanomaterials and neutrophils are currently well-recognized for scavenging invaders and modulating the innate immune system. Various engineered nanomaterials have been developed and have demonstrated a beneficial role in neutrophil-related diseases, such as bacterial infection, acute lung injury, vascular inflammation, autoimmune diseases and cancer [192,193]. Therefore, understanding how nanomaterials interact with neutrophils can help in developing safer and more effective nanomedicine applications. Neutrophils can be recruited during inflammation using different adhesion molecules such as P- and E-selectins via adhesion, crawling, tethering, rolling, and transmigration to accumulation at the inflammatory site [194]. Once neutrophils are activated and recruited at inflammation site, they can eliminate pathogens through processes like the release of neutrophil extracellular traps (NETosis), degranulation, etc. [195]. Researchers have designed various nanomaterials to alleviate inflammation and modulate the inflammatory microenvironment by specifically targeting neutrophils in various disease models, suggesting its significant role in the pathogenesis of various inflammatory diseases [31,196,197,198]. The mechanisms of nanomaterial-based immunomodulation include modulating neutrophil migration, neutrophil-based drug delivery, depleting inflammation-related neutrophils, neutrophil biomimetic techniques etc. [15].

Neutrophils play a primary role in a variety of inflammatory diseases by adhering to the vascular endothelium and migrating into lesional tissue during the inflammation process [199]. Therefore, hampering the neutrophil migration cascade to alleviate inflammation represents a promising strategy. Researchers have reported inhibition of inflammation-related neutrophil migration using different nanoformulations. Park et al. reported neutrophil-related immune response reprogramming through neutrophil internalization in a traumatic primary spinal cord injury (SCI) mouse model via administration of PLGA nanoparticles [200]. The results indicated regeneration and functional recovery with restoration of a proregenerative microenvironment through a reduction in neutrophil accumulation (four-fold) at the injury site after treatment with PLGA nanoparticles [200]. In addition, the altered migration process of neutrophils demonstrated a significant increase in the number of neutrophils and accumulation in the spleen. Similar strategies have also been employed in acute lung injury and experimental autoimmune encephalomyelitis models, which demonstrated a significant decrease in disease severity, with migration of neutrophils to the spleen rather than inflammatory lesions [197,201]. Moreover, these results report the capability of nanoparticles to modulate the migration patterns of neutrophils for the amelioration of inflammatory diseases.

Another mechanism of nanomaterial-mediated immunomodulation involving neutrophils includes the depletion of inflammation-related neutrophils from the inflammatory region. In order to attain this, different strategies such as the depletion of local neutrophils and the suppression of neutrophil activity and proliferation are being utilized to alleviate local inflammation severity. To attempt this, doxorubicin (DOX)-conjugated pH-responsive albumin nanoparticles have been developed to specifically target and deplete activated neutrophils [196]. These nanoparticles are internalized by neutrophils and have demonstrated therapeutic effects in models of acute lung inflammation and ischemic stroke. In patients with coronavirus (COVID-19), neutrophil-related inflammation is exacerbated by the release of cell-free DNA (cfDNA) and pro-inflammatory cytokines. A recent study showed that DNase-1-coated polydopamine-poly(ethylene glycol) nanoparticles could suppress neutrophil activity and mitigate the resulting cytokine storm in a mouse model of sepsis [198]. This treatment resulted in a significant increase in survival rates among the treated groups, accompanied by a notable reduction in neutrophil counts and levels of various inflammatory cytokines.

Additionally, neutrophils can function as transport carriers for therapeutics, akin to the hitchhiking strategies employed by macrophages. In an earlier investigation, albumin nanoparticles loaded with TPCA-1 (2-[(aminocarbonyl)-amino]-5-(4-fluorophenyl)-3-thiophenecarboxamide), an NF-κB inhibitor, were developed for treating acute lung inflammation in mice [202]. The findings revealed a substantial decrease in inflammatory markers, including reduced neutrophil infiltration and cytokine release, as well as the prevention of lung edema. Another study utilized poly-l-lysine (DGL) crosslinked dendrigraft nanoparticles to deliver catalase in a mouse model of cerebral ischemia [203]. Targeting ligands, such as Pro-Gly-Pro (PGP), modified the nanoparticles, enhancing their affinity for neutrophils and resulting in decreased infarction volume in the treated group. Moreover, a new nanomaterial-mediated strategy to deliver therapeutics through neutrophil hijacking to inflammatory lesions has been developed for the inhibition of ROS-mediated apoptosis, which might be the underlying mechanism.

### 4.3. Interactions Between Nanomaterials and Lymphocytes (T- and B-Cells)

A variety of nanomaterial-based platforms have been developed for the management of inflammatory diseases, autoimmune diseases, etc., to target these cellular components (T and B lymphocytes) via modulation of the immune system. Understanding how nanomaterials interact with these cells can aid in the development of advanced therapeutic strategies, such as targeted drug delivery, vaccines, and immunotherapies. The mechanisms of action include the induction of T-cell-mediated immune, autoreactive T lymphocyte depletion and the modulation of immune responses related to B-cells.

#### 4.3.1. Nanomaterial-Based Immunomodulation of T Lymphocytes

T-cells are one of the main components of the adaptive immune system and include CD8^+^ cytotoxic T lymphocytes (CTLs), CD4^+^ helper T lymphocytes (Ths), regulatory TT lymphocytes (Tregs) and memory TT lymphocytes (Tms). CTLs represent the main effector cells of the adaptive immune system, while Th cells modulate different immune responses and activate CTL cells and B lymphocytes through secretion of cytokines. Conversely, Treg cells inhibit the activity of effector cells by acting as negative modulators of the immune system. One of the mechanisms that directs T lymphocyte immunomodulation through nanomaterials includes the induction of T-cell-related immune tolerance. One of the major concerns in the pathogenesis of various inflammatory diseases is autoreactive immune response. Therefore, strategies such as relevant antigen delivery to induce immune tolerance and the re-establishment of immune tolerance have been considered effective treatment methods for inflammatory diseases. In this context, PLGA nanoparticles encapsulated with antigens were employed to treat Th2-mediated allergic airway inflammation and T1/T17-mediated autoimmune disease by inducing Th2 immune responses both prophylactically and therapeutically [204,205].

In order to regulate peripheral immunological tolerance, Tregs are another important cell that inhibit the function of other immune cells such as macrophages, DCs, CD4^+^ and CD8^+^ T-cells using various mechanisms [206]. Tregs play a critical role in inhibiting T-cell survival and proliferation by secreting immunosuppressive cytokines such as IL-10 and TGF-β, reducing IL-2 levels, and preventing T-cell activation and the maturation of antigen-presenting cells (APCs). Researchers are investigating nanotechnology-based immunomodulatory approaches to enhance the function of Tregs by promoting their expansion. This modulation can significantly boost anti-inflammatory responses and impede the progression of autoimmune diseases. For instance, specific subsets of Tregs, like FoxP3^+^ Treg cells, are employed to induce immune tolerance by interacting with various components of the immune system. Nanomaterial platforms have been utilized for Treg cell expansion in various autoimmune disease models [207]. Clemente-Casares et al. developed peptide-coated nanoparticles linked to MHC class II (pMHCII) molecules aimed at treating autoimmune conditions. The systemic delivery of these relevant peptides triggered the differentiation and expansion of antigen-specific regulatory CD4^+^ T-cell type 1 (TR1)-like cells in models such as experimental autoimmune encephalomyelitis (EAE) and collagen-induced arthritis (CIA) [208]. The use of nanomaterials to restore established autoimmune conditions activated a cascade of cellular interactions, leading to the release of transcription factors like T-bet and c-Maf, which regulated the production of IL-10, IFN-γ, and IL-21, thereby enhancing the therapeutic effects of pMHC nanoparticles [208]. In another study, PLGA nanocarriers decorated with the murine allergen ovalbumin (OVA) targeted specialized tolerogenic liver sinusoidal endothelial cells (LSECs). [209]. These cells, capable of generating Tregs, significantly alleviated airway inflammation by promoting the differentiation of Foxp3^+^ Treg cells in animal models. Additionally, Ohno and his team demonstrated that a nanoparticle–curcumin system increased the expansion of CD4^+^ Foxp3^+^ regulatory T-cells for treating inflammatory bowel diseases (IBD). This nanoparticle–curcumin platform reduced body weight loss, improved histological scores for colitis, and enhanced disease activity indices, while promoting mucosal permeability [210]. Furthermore, another study showed that AuNPs coated with a potent TLR inhibitor hexapeptide significantly boosted the population of Tregs by inhibiting the activation of TLR signaling pathways (TLR2, TLR3, TLR4, and TLR5), with reduced inflammation, infiltration and promotion of neutrophil apoptosis in an LPS-induced acute lung injury model [211]. Maldonado et al. demonstrated prevention of systemic immunosuppression using peptide antigens and rapamycin-loaded tolerogenic PLGA NPs. The tolerogenic-decorated nanoparticles inhibited T-cell activation by generating Tregs and antigen-specific tolerance induction in multiple animal models [170]. Moreover, the immune tolerance was maintained without systemic immunosuppression for as long as 111 days.

Another approach that includes the depletion of autoreactive T lymphocytes has been utilized for a variety of inflammatory diseases like acute inflammation and rheumatic arthritis. Several nanoparticle platforms have been utilized for depleting lymphocytes using different strategies viz., cell signaling inhibition, suppression of cell receptors, and cytotoxic drug delivery. Özcan et al. developed AuNPs loaded with methotrexate (MTX), an antiproliferative drug, and demonstrated superior anti-inflammatory efficacy with significantly reduced γδ T and CD4^+^ T-cell count and inhibition of skin hyperplasia in an imiquimod-induced psoriasis mouse model [212]. In recent years, the Hedgehog (Hh) signaling pathway represents a therapeutic target owing to its critical role in T-cell activation for the treatment of inflammatory diseases [213]. Researchers have designed anti-CD4 F(ab′) antibody fragment-decorated polymeric nanoparticles loaded with a Hh inhibitor to CD4^+^ T-cells, eggmanone (Egm), and have showed a reduction in helper T-cell activation and cytokine secretion through significant inhibition of CD4^+^ T-cell responses [50]. Wu et al. developed superparamagnetic iron oxide (SPIO) nanoparticles for demonstrating efficient control of lung inflammation using anti-ST2-conjugated nanoparticles by suppressing innate lymphoid cell 2 (ILC2) receptor, tumorigenicity 2 (ST2)-led suppression [214]. NPs conjugated with the anti-ST2 antibody reduced the ability of ILC2 to produce IL-5 and IL-13 via inhibition of CD4^+^ T-cell differentiation and expansion to alleviate lung inflammation in the murine model of asthma [214]. Taken together, the immunomodulation of T-cells using nanomaterials is cutting-edge research in immunotherapy. This approach leverages the unique properties of nanomaterials to manipulate the immune response, particularly focusing on T-cells.

#### 4.3.2. Nanomaterial-Based Immunomodulation of B Lymphocytes

B lymphocytes (B-cells) are effector cells of the adaptive immune system, which differentiate into plasma cells and secrete antibodies after the binding of antigens to B-cell receptors (BCR) [215]. The types of nanomaterials being employed for B-cell immunomodulation include nanoparticles, liposomes, dendrimers, carbon dots and quantum dots. The mechanisms of immunomodulation include targeted delivery of immunomodulatory agents, antigen presentation, gene editing, cytokine delivery and immunosuppression. Nanomaterials can be engineered to specifically deliver immunomodulatory drugs to B-cells, enhancing treatment efficacy while minimizing side effects. Nanomaterials can also present antigens to B-cells, promoting their activation and proliferation, which is useful in vaccine development and cancer immunotherapy. Delivery of gene-editing tools like CRISPR/Cas9 to B-cells using nanomaterials can modify genes involved in B-cell function and disease. Nanomaterials can be used to deliver cytokines directly to B-cells, modulating their activity and promoting a desired immune response. For conditions requiring reduced immune activity, nanomaterials can deliver immunosuppressive agents specifically to B-cells. Moreover, the immunomodulation of B-cells using nanomaterials is an emerging field with significant potential in treating various immune-related conditions.

Autoreactive B-cells with the ability to secrete autoantibodies have been reported in the pathogenesis of several inflammatory diseases. Currently, the nanoparticle-based strategies employed for B lymphocyte immunomodulation focused on direct targeting of autoreactive B-cells and their inhibition/depletion for inflammatory disease management. Bednar et al. developed Siglec-engaging tolerance-inducing antigenic liposomes (STALs), which co-displayed synthetic citrullinated antigens (CCP STALs) and CD22, an immune inhibitor for the treatment of rheumatoid arthritis (RA) [216]. The findings of this research indicated that tolerance to citrullinated antigens was successfully induced in a mouse model through the inhibition of pathogenic factors by CCP STALs, along with the generation of anti-citrullinated protein antibodies (ACPAs) from the memory B-cells of RA patients. Additionally, another study revealed that PLGA nanoparticles, decorated with synthetic citrullinated peptides and complement-activating lytic peptides, effectively depleted autoreactive B-cells through targeted treatment for RA. The developed nanosystem significantly reduced B-cell-derived ACPA production in peptide seropositive RA patients [217]. This approach of B-cell inhibition/depletion has also been employed for the treatment of central nervous system (CNS) autoimmune diseases using PLGA nanoparticles. A recent study introduced a nanotechnology-driven drug delivery system that utilizes cell-mediated mechanisms. In this system, nanoparticles are loaded with T-cells that target myelin antigens and are combined with an anti-CD20 monoclonal antibody [218]. This innovative delivery method effectively reduced B-cell populations in both the spleen and brain within an animal model of multiple sclerosis (MS) and experimental autoimmune encephalomyelitis. Nevertheless, this approach effectively ameliorated the disease course and pathology. Overall, the interaction between nanomaterials and lymphocytes is complex and influenced by multiple factors, including the physical and chemical properties of the nanomaterials and the biological environment. By comprehensively understanding these interactions, researchers can design nanomaterials that effectively harness lymphocyte functions for therapeutic purposes while minimizing adverse effects. The ability to precisely manipulate B-cell activity opens up new avenues for treating a wide range of immune-related conditions.

## 5. Case Studies of Nanomaterial-Mediated Immunomodulation

Case studies of immune modulation by nanomaterials highlight the versatility and potential of these advanced materials in various therapeutic contexts. From cancer immunotherapy and autoimmune disease treatment to infectious diseases, vaccination and transplant tolerance, nanomaterials offer innovative solutions for enhancing therapeutic outcomes. One notable example involves the use of AuNPs in cancer immunotherapy. In a clinical trial, nanocomposite tectons (NCTs) using AuNPs conjugated with tumor-specific antigens were administered to patients with melanoma. The AuNPs facilitated the efficient delivery and presentation of these antigens to DCs, significantly enhancing the activation of T-cells and promoting a robust anti-tumor immune response.

### 5.1. Cancer Immunotherapy

Despite its promise, cancer immunotherapy faces several significant challenges that limit its efficacy for many patients [24,191,219,220]. Moreover, inefficient uptake and presentation by APCs, suppression of APC function, immunosuppressive environment formation, abnormal cellular metabolism, and extracellular matrix (ECM) fibrosis challenge conventional cancer immunotherapy. Therefore, there is a pertinent requirement for the development of newer approaches and strategies for cancer immunotherapy. Nonetheless, the combination of nanomedicine and immunotherapy has garnered more attention in the last decade. A comprehensive list of different nanomaterials being utilized for the treatment of various cancers is provided in Table 4.

**Figure 7:**
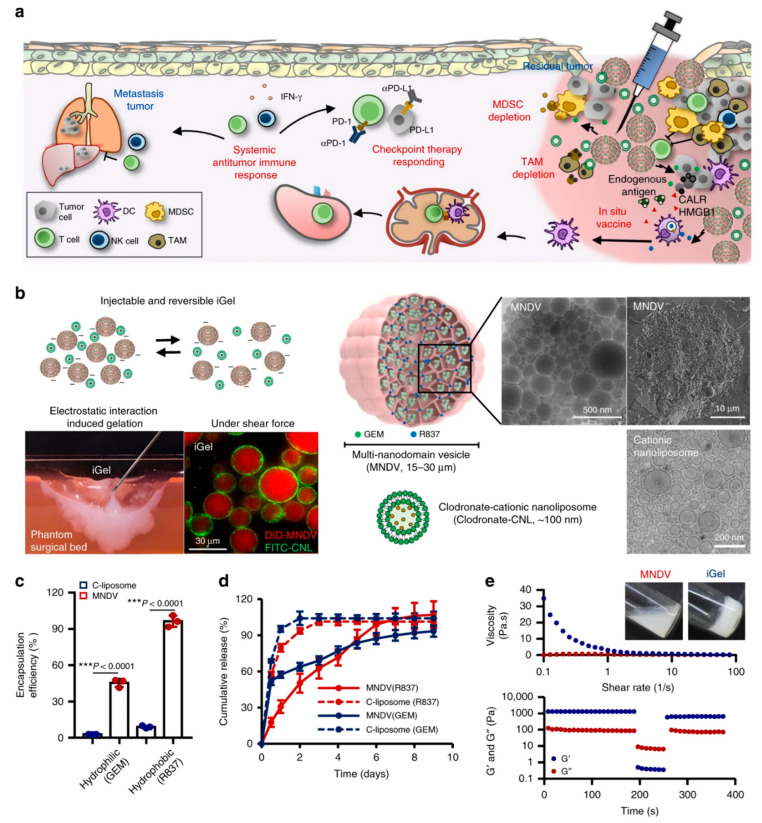
Immunomodulatory multi-domain nanogel (iGel) loaded with gemcitabine (GEM) and hydrophobic imiquimod (R837) for the prevention of tumor metastasis and recurrence in post-surgical treatment. (**a**) Schematic depicting construction of iGels loaded with GEM and R837 for TAM and MDSC depletion for reverting the immunosuppressive environment and exerting an anti-tumor response, (**b)** **Left**: A photograph showing easy injectability of iGel and fluorescent imaging confirming DID loaded MNDVs interconnected by FITC-labeled CNLs. **Right**: MNDVs and CNL schematics with electron cryomicrographs, (**c**) encapsulation efficiency of hydrophilic GEM and hydrophobic R837 drug loading in MNDV and C-liposomes (**d**) drug release kinetics from liposomes and MNDVs, and (**e**) rheological properties of iGels. Reproduced from ref. [222] with permission from Nature Publishing Group. This work is licensed under a Creative Commons Attribution 4.0 (CC BY) International License.

Currently, liposomes are widely used nanomaterials that function as immunotherapeutic nanosystems for clinical applications, due to their low toxicity and immunogenicity. Examples of FDA-approved liposomes include Lipovaxin MM, tecemotide, Lipo-MERIT, and iscomatrix. In addition to liposomes, various other nanomaterials are undergoing clinical trials, having been deemed safe for human use. For instance, the cancer vaccine Oncoquest-L, which is derived from a patient’s own cancer cell extracts, is currently in phase II clinical trials (NCT02194751). Furthermore, two cholesteryl pullulan-based cancer vaccines, CHP-NY-ESO-1 and CHP-HER2, are being tested in clinical trials (NCT00291473) alongside the OK-432 adjuvant, demonstrating effectiveness in patients with esophageal cancer [221]. Immunomodulatory nanomaterials offer anti-tumor effects via two ways, namely the activation of antigen-presenting cells (APCs) and regulation of the immunosuppressive tumor microenvironment. In an earlier report, Song and co-workers developed immunomodulatory nanogels loaded with both hydrophilic and hydrophobic drugs to overcome the limitation of pro-tumoral TME reprogramming to anti-tumoral immune niches [222]. Herein, an immunomodulatory multi-domain nanogel (iGel) is formed using positively charged nanoliposomes and negatively charged non-concentric multi-nanodomain vesicles (MNDVs) through electrostatic interactions. The sustained and extended release of drugs inhibited the metastasis and recurrence of tumors post-surgery. The combination therapy combining checkpoint blockade therapies and immunotherapies reverted the immunosuppressive microenvironment of TME, depleted the TAMs and MDSCs, and exerted a strong anti-tumor response, as shown in Figure 7. PLGA nanoparticles was also used as immunostimulatory adjuvants for the delivery of TLR7/8 agonists to boost DC activation [148]. The results indicated the migration of nanoparticles to draining lymph nodes by triggering the activation and expansion of DCs. The triggered activation of DCs resulted in antigen-specific CD8^+^ T-cell expansion and enhanced CTL response, which showed significant therapeutic efficacy in renal cell carcinoma, melanoma, and bladder tumor models. Furthermore, Gargett and co-workers utilized Lipovaxin MM, a tumor vaccine targeting DCs that is currently in the phase I clinical trial stage (NCT01052142), for immunotherapeutic applications [223]. Lipovaxin-MM, which comprises an Ab fragment target, specifically a DC-specific intercellular adhesion molecule, was tolerated well in metastatic melanoma patients.

**Table 4:** A representative list of different immunomodulatory nanomaterials utilized in cancer immunotherapy.

Type of Nanomaterial	Type of Cancer	Mechanism	Findings	References
Dendrimers	Prostate cancer	T-cell activation via antigen presentation	Enhanced T-cell activation, decreased metastasis	[224]
Carbon nanotubes	Colon cancer	Immune activation and cytokine release	Enhanced cytotoxicity, improved immune response	[225]
Silver nanoparticles	Lung cancer	ROS generation and immune activation	Induced apoptosis, enhanced immune response	[144]
Nanogels	Gastric cancer	Immune modulation through cytokine delivery	Improved drug delivery, increased apoptosis	[191]
Graphene oxide	Breast cancer	Immune activation and cytokine modulation	Enhanced immune response, reduced tumor size	[226]
Zinc oxide nanoparticles	Lung cancer	ROS generation and immune activation	Induced apoptosis, enhanced immune response	[227]
Chitosan nanoparticles	Colon cancer	Immune modulation and biocompatibility	Enhanced immune response, reduced tumor size	[228]
Calcium phosphate nanoparticles	Ovarian cancer	Immune modulation through drug delivery	Improved drug delivery, induced apoptosis	[229]
PLGA nanoparticles	Breast cancer, ovarian cancer	Controlled release and immune activation	Enhanced immune response, improved efficacy	[51,230]
Mesoporous silica nanoparticles	Brain cancer, skin cancer	High surface area for immune adjuvant loading	Targeted immune activation, reduced side effects	[70]
Liposomes	Skin cancer, colorectal cancer	Encapsulation of immune adjuvants	Improved immune response, increased survival rates	[47,48,49]
Magnetic nanoparticles	Skin cancer	Magnetic targeting and immune activation	Enhanced immune response, improved MRI	[40,65,66]
Polymer nanoparticles	Ovarian cancer	Sustained release of immune modulators	Improved efficacy, enhanced immune response	[230]
Iron oxide nanoparticles	Liver cancer	Magnetic targeting and immune modulation	Improved imaging, targeted immune therapy	[231,232]
Quantum dots	Pancreatic cancer	Immune targeting via fluorescence	Enhanced imaging, targeted immune response	[233]
Silica nanoparticles	Liver cancer	High drug-loading capacity with immune modulation	Improved drug loading, targeted immune therapy	[234]

### 5.2. Infectious Disease Treatment

Immunomodulatory nanomaterials are revolutionizing the treatment of infectious diseases by enhancing the delivery and efficacy of immunomodulating agents. Their versatility allows for multifunctional platforms that simultaneously deliver antimicrobial agents and immune modulators, offering promising strategies against infections like HIV, influenza, Ebola, and tuberculosis [167,235,236].

#### 5.2.1. Viral Infection Treatment

Nanotechnology-based approaches open new avenues for the development of a novel class of anti-infective medications. Earlier research reports have supported the positive outcomes of nanomaterial-based vaccine development against influenza viruses and other infectious diseases [237,238]. Furthermore, vaccine formulations based on nanotechnology greatly enhance immune protection and their immunogenicity has been drastically enhanced by delivering antigens and adjuvants via nanomaterials. In recent years, virosomes (non-infectious artificial viruses) and liposome-derived nanovaccines have attracted much attention for targeting viral infections with excellent potency in the body [27]. Until now, two nanostructured vaccines, Inflexal V and Epaxal, have been FDA approved for the treatment of hepatitis A and influenza [239]. Apart from these nanomaterials, metallic nanosystems, especially AuNPs, have also exhibited promising antiviral activities against different types of viruses, such as influenza, norovirus, poliovirus, dengue virus, HIV, hepatitis, herpes simplex virus, chikungunya virus, human adenoviruses, coronavirus, coxsackievirus, rubeola virus, etc. [240]. The developed nanomaterials have modulated both innate (DCs, macrophages) and adaptive immune responses (B-cells and T-cells) to demonstrate an antiviral response. Orlowski et al. utilized gold and silver NPs modified with tannic acid as potential stimulators for the activation of DCs against herpes simplex virus (HSV) [79]. The modification in silver and gold NPs induced the maturation of DCs via internalization by the antigen-presenting cells without being degraded by lysosomes. In another study, gold nanorods stimulated the innate immune response to inhibit respiratory syncytial virus (RSV) infections in treated lung tissues by indicating gene expression of multiple antiviral networking profiles related to TLR and NLR pathways [241]. Furthermore, Gomez et al. reported initiation and enhancement of downstream inflammatory cascade pathways via the release of the pro-inflammatory cytokines IL-1 *β* and IL-18 as a mechanism for activating the innate antiviral immune response [80]. The ability of T-cells and cytokines to combat viral pathogens effectively mitigates the negative effects often associated with conventional agonists or antagonists targeting cytotoxic T-cells and cytokines. For example, virus-like nanoparticles conjugated with RSV glycoproteins were created to enhance T-cell responses against the virus [242]. This conjugated glycoprotein mixture contained fusion glycoproteins, which led to an increase in CD4^+^ and CD8^+^ T-cell levels, as well as Th2 cytokines (such as IL-5 and IL-13) and IL-4-producing cells, while reducing levels of IFN-γ. This modulation suppressed the production of pro-inflammatory cytokines and decreased T-cell infiltration, resulting in long-lasting immunity against RSV without excessive inflammation in lung tissues.

#### 5.2.2. Bacterial Infection Treatment

Immunomodulatory nanomaterials are at the forefront of innovative treatments for bacterial infections, offering a novel approach that harnesses the power of the immune system through specific interactions with immune cells, specific targeting of bacteria, and an enhanced ability to combat infections [28,236]. Several research groups reported different nanomaterials with enhanced anti-infection outcomes via delivering antigens and/or adjuvants [243,244]. Vetro et al. developed T-helper peptide OVA-loaded Au NPs modified with pneumococcal capsular polysaccharide antigens to combat pneumococcal infections [167]. The glycoconjugate vaccine elicited a specific IgG antibody-dependent immune response against Streptococcus pneumoniae in a mouse model. Similarly, Hu et al. created a toxin nanosponge by fusing PLGA nanoparticles with red blood cell (RBC) membrane vesicles, aimed at neutralizing a broad range of potent virulence factors known as pore-forming toxins (PFTs) with a high affinity [245]. In this approach, PLGA nanoparticles served as a core to stabilize the RBC membrane shell, thereby extending the circulation time of the nanomaterials in the bloodstream for toxin elimination. The PLGA-based nanosponge effectively neutralized staphylococcal α-hemolysin (a model PFT) by diverting the intended target cells, leading to macrophage-mediated ingestion in toxin-challenged mice. Additionally, this formulation provided robust protection against various bacterial infections caused by four PFTs, including listeriolysin O from L. monocytogenes, streptolysin O from Group A Streptococcus, melittin, and α-hemolysin from methicillin-resistant Staphylococcus aureus (MRSA) [246]. Besides RBC membrane vesicles, bacterial outer membrane vesicles (OMVs) secreted by E. coli have also been used to coat Au NPs, providing another favorable option for constructing biomimetic nanoparticle platforms [247]. The OMV-NPs activated DCs and rapidly generated a stronger immune response mediated by T-cells and B-cells, demonstrating a synergistic effect of OVA and Au NPs against bacterial infections.

### 5.3. Allergic and Autoimmune Disease Treatment

In autoimmune diseases, wherein the immune system attacks the body’s own tissues mistakenly, immunomodulatory nanomaterials offer a means to restore immune tolerance. These nanomaterials can be engineered to carry tolerogenic agents that promote regulatory T-cell expansion or inhibit the secretion of pro-inflammatory cytokines such as IL-10 or TGF-beta [248]. The engineered nanomaterials help to recalibrate the immune system, reducing the pathological immune responses that characterize autoimmune conditions. In order to alleviate this issue, developed tolerogenic nanomaterials have been reported beneficial for the treatment of food allergy, anaphylaxis, systemic lupus, erythematosus, asthma, rheumatoid arthritis, and other allergic and autoimmune diseases [209,249,250]. The treatment approach includes molecules that target and enhance naturally occurring immunoregulatory pathways as well as the delivery of disease-relevant autoantigens to professional APCs.

Nanomaterial-based therapies have demonstrated potential in both preclinical and clinical settings for treating various autoimmune disorders, including rheumatoid arthritis (RA), multiple sclerosis (MS), and systemic lupus erythematosus [251,252]. The development of novel strategies targeting disease-mediating cells offers a promising direction for treating autoimmune diseases [253]. These new approaches focus on modulating total immunosuppression, which can lead to adverse long-term side effects. Researchers have utilized altered peptide ligands (APLs) of autoantigens in animal models of autoimmune diseases to induce antigen-specific tolerance. This strategy aims to modulate the functions of autoreactive T-cells by leveraging the specific interactions between T-cell receptor–peptide MHC complexes (TCR–pMHC) to promote immunotolerance. However, this method has limitations, including the inability to accurately predict the immune response to specific APLs, and the suppression of autoreactive T-cells can negatively impact systemic immunity. To address these challenges, a new strategy involves delivering tolerogenic substances to T-cells, facilitating the transformation of naive CD4^+^ T lymphocytes into effector T-cells (Teff) or regulatory T-cells (Treg). These transformed cells can recognize self-peptides on antigen-presenting cells (APCs) with assistance from concurrent stimulatory or inhibitory signals in their microenvironment. For instance, Park and colleagues utilized PLGA nanoparticles loaded with the inflammatory cytokine interleukin-6 (IL-6) for the specific targeting of CD4^+^ T-cells [254]. This targeted approach resulted in increased RORγT expression and inflammatory activity due to inappropriate Th17 cell function. In contrast, nanoparticles containing the tolerogenic cytokine leukemia-inhibitory factor (LIF) enhanced the expression of Foxp3^+^, promoting the development and immune tolerance of Treg cells. The ability to precisely target and modify immune components makes immunomodulatory nanomaterials a significant advancement in the treatment of autoimmune and allergy diseases. These nanomaterials enable more balanced, regulated immune responses, in contrast to traditional treatments that frequently result in widespread immunosuppression and adverse effects. These materials have the potential to completely transform medical practices as research progresses, providing more potent, long-lasting, and patient-specific treatments for immune-related ailments.

### 5.4. Wound Healing

The complexity of wound-healing has increased, necessitating the use of sophisticated biomaterials with immunomodulatory and antimicrobial properties. Because of their low effectiveness and growing resistance, traditional antibiotic treatments frequently fail. GCP hydrogels based on glycyrrhizic acid, in conjunction with copper and polyphenols, exhibit enzyme-mimetic activity (SOD and CAT) for ROS scavenging and regulated Cu^2+^ release, hence augmenting angiogenesis and antibacterial activity [255]. These hydrogels hold promise for intelligent wound care since they also alter macrophage activity to encourage tissue regeneration. Black phosphorus nanosheets and zinc oxide are combined into a three-dimensional network in BP@ZnO-Gel/SA hydrogel, another novel technology that offers superior mechanical and biological properties, in addition to the efficient photothermal and photodynamic killing of microbes [256]. This hydrogel accelerates wound healing, encourages angiogenesis, and improves immunological control. Together, these cutting-edge nanomaterials and hydrogel systems offer multipurpose platforms that integrate antimicrobial, anti-inflammatory, ROS-regulating, and immunomodulatory qualities, greatly improving wound-healing results and providing encouraging approaches to the treatment of chronic and infected wounds.

### 5.5. Bone Regeneration and Repair

Effective bone regeneration requires not only the stimulation of osteogenesis, but also the modulation of the inflammatory microenvironment. The natural bone interface is also mimicked by hierarchical intrafibrillar mineralized collagen (HIMC) scaffolds, which promote endogenous bone regeneration by polarizing macrophages toward the M2 phenotype and encouraging the secretion of interleukin-4 (IL-4), which in turn stimulates the osteogenic differentiation of mesenchymal stem cells (MSCs). Effectively attracting CD146^+^STRO-1^+^ MSCs and promoting neo-bone formation, HIMC’s effects are greatly diminished when macrophages are reduced or IL-4 is neutralized. In a different strategy, gold nanocages coated with cytokine-receptor-rich macrophage membranes and loaded with resolvin D1 form biomimetic anti-inflammatory nanocapsules (BANCs), which function in a phased manner. By coordinating the immunomodulatory effects with the bone-healing timeline, they promote tissue repair in femoral bone defects by first neutralizing inflammatory cytokines and subsequently inducing M2 macrophage polarization under near-infrared irradiation [257].

In acidic lysosomes, calcium nervonate nanoparticles, a unique dual-functional nanoplatform, break down to liberate nervonic acid and calcium ions. Calcium promotes MSC osteogenic development, whereas nervonic acid reduces activated macrophage inflammatory responses. Studies conducted in vivo demonstrate their synergistic effect and validate their better bone regeneration potential when compared to individual components [258]. Together, these cutting-edge nanomaterials show off the effectiveness of immunomodulation in bone tissue engineering through the regulation of ROS, cytokine neutralization, macrophage polarization, and osteoinductive signaling. Thus, these studies set up the basis for next-generation osteoimmunomodulatory therapies by providing innovative and effective methods for restoring bone abnormalities induced by inflammation or trauma.

## 6. Clinical Trials

Clinical trials of immunomodulatory nanomaterials represent a cutting-edge frontier in the treatment of various diseases, capitalizing on the unique properties of nanomaterials to enhance therapeutic efficacy and safety. An update on clinical trials using different immunomodulatory nanomaterials for disease treatment is presented in Table 5. One notable area of focus has been in cancer treatment, where nanoparticles are used to deliver checkpoint inhibitors and other immunotherapeutic agents directly to the tumor microenvironment. Clinical trials have shown that such targeted delivery can significantly enhance the anti-tumor immune response while reducing systemic side effects. For instance, a trial involving NCT nanoparticles encapsulating the checkpoint inhibitor pembrolizumab demonstrated improved tumor targeting and a higher rate of tumor shrinkage compared to traditional administration methods. In the realm of infectious diseases, clinical trials are exploring the potential of nanomaterials to enhance vaccine efficacy and antiviral treatments. Nanoparticle-based vaccines are being tested for their ability to elicit stronger and longer-lasting immune responses against pathogens like influenza, HIV, and COVID-19. These trials are particularly promising because nanomaterials can be engineered to mimic the size and shape of viruses, thereby improving antigen presentation and stimulating a more robust immune response. Additionally, clinical trials involving liposomal formulations of antiviral drugs aim to improve drug delivery and reduce the dosage required, thereby minimizing side effects and overcoming issues related to drug resistance.

Autoimmune diseases are another critical focus of clinical trials involving immunomodulatory nanomaterials. Trials are investigating the use of nanoparticles to deliver immunosuppressive agents directly to affected tissues, aiming to reduce systemic immunosuppression and its associated risks. For example, a clinical trial testing the efficacy of nanoparticles loaded with methotrexate for treating rheumatoid arthritis NCTs has shown that targeted delivery can significantly alleviate joint inflammation and pain with fewer side effects compared to conventional therapy. Such approaches hold promise for more effective and safer management of autoimmune conditions by precisely modulating the immune response. Furthermore, clinical trials are exploring the use of nanomaterials for modulating the immune system in the context of organ transplantation and chronic inflammatory diseases. For instance, nanoparticle-based delivery of regulatory T-cells or cytokines that promote immune tolerance is being tested to prevent graft rejection and manage conditions like inflammatory bowel disease. These trials are crucial for understanding the long-term safety and efficacy of nanomaterial-based therapies in complex immune-related conditions.

Overall, clinical trials of immunomodulatory nanomaterials mark a significant advancement in the field of medicine. These trials underscore the potential of nanotechnology to revolutionize disease treatment by providing more targeted, effective, and safer therapeutic options. As the body of evidence from these trials continues to grow, it is expected that nanomaterials will play an increasingly important role in the clinical landscape, offering new hope for patients with challenging medical conditions. Continued research and development in this area will be crucial to fully realize the potential of nanomaterials in clinical practice, paving the way for more innovative and effective treatments.

## 7. Challenges and Future Prospects

Despite providing tremendous promise in revolutionizing disease treatment, immunomodulatory nanomaterials also face several challenges that must be addressed to realize their successful translation from the laboratory to clinical application. One of the primary challenges is the complexity of interactions between nanomaterials and the immune system. While nanomaterials can be engineered to enhance immune responses, there is also a risk of triggering unintended immune reactions or inflammatory responses. Understanding how different types of nanomaterials interact with immune cells and tissues is crucial for predicting and managing potential immunotoxicity or adverse immune reactions. In this context, systematic investigation of the intrinsic immunomodulatory properties of widely used nanocarrier materials, independent of their therapeutic payloads, is essential. Standardized assessment frameworks integrating physicochemical characterization with in vitro immune profiling and in vivo validation are needed to define whether carrier-induced immune effects contribute to therapeutic efficacy and safety. This challenge is further compounded in composite nanoplatforms carrying active therapeutic agents, where the observed immunomodulatory effects may arise from the nanocarrier itself, the encapsulated drug, or their combined interaction. To disentangle these contributions, preclinical evaluation should incorporate appropriate controls such as unloaded nanocarriers, free drug formulations, and non-encapsulated physical mixtures, with equivalent doses and exposures. Such systematic study designs are critical for distinguishing carrier-driven, drug-driven, and synergistic immune effects, thereby enabling more accurate interpretation of therapeutic efficacy and safety.

Another significant challenge is the regulatory landscape surrounding nanomaterials in healthcare applications. Many countries lack specific regulations tailored to nanomedicine, leading to uncertainties in approval processes and safety assessments. Regulatory agencies worldwide are grappling with how to classify and assess the safety of nanomaterials, which often exhibit unique physicochemical properties that conventional regulatory frameworks may not adequately address. Harmonizing regulatory guidelines and developing standardized protocols for evaluating the safety and efficacy of immunomodulatory nanomaterials are essential steps towards their clinical adoption. Additionally, the scalability and reproducibility of nanomaterial synthesis pose challenges in clinical applications. Another critical challenge is achieving consistent and scalable production of immunomodulatory nanomaterials. Manufacturing processes must be standardized to ensure uniformity in particle size, shape, and surface characteristics, which can influence their biological interactions and therapeutic outcomes. Developing robust and scalable manufacturing processes is therefore essential for overcoming these barriers to clinical translation. Furthermore, there are challenges related to the long-term stability and biocompatibility of immunomodulatory nanomaterials. Nanoparticles and other nanoscale materials may undergo degradation or aggregation over time, potentially affecting their therapeutic efficacy and safety. Ensuring the stability of nanomaterials in biological environments and minimizing their accumulation in non-target tissues are ongoing challenges in nanomedicine research.

Although physicochemical properties such as size, shape, and surface chemistry and the dynamic formation of the protein corona are recognized as key determinants of nanomaterial biodistribution and immune interactions, the metabolic fate and degradation pathways of many nanomaterials in vivo remain poorly understood. Nanomaterial transformation processes, including biodegradation, dissolution, and clearance, may substantially alter immune recognition, persistence, and the downstream immunomodulatory effects. Currently, there is a lack of standardized and sensitive methodologies to systematically investigate these processes and their immunological consequences in vivo. Developing advanced analytical, imaging, and longitudinal in vivo models to track nanomaterial transformation alongside immune responses will be critical for improving safety assessment, predictive immunotoxicology, and rational nanomaterial design.

Advances in nanomaterial design, surface modification, and formulation strategies are needed to enhance their biocompatibility and stability, thereby improving their clinical utility and safety profile. Other significant challenge is ensuring the safety of nanomaterials in clinical settings. Despite their engineered properties, there are concerns about their potential toxicity, immune reactions, and long-term effects on human health. Rigorous testing and characterization are essential to understand how nanomaterials interact with biological systems and to mitigate any adverse effects. Additionally, the cost-effectiveness of nanomaterial production and clinical translation remains a barrier. Developing scalable synthesis methods and optimizing production costs are essential to make these advanced therapies accessible to a broader patient population. Addressing these challenges through multidisciplinary research efforts and collaborative initiatives will be crucial for realizing the full potential of immunomodulatory nanomaterials in transforming healthcare and disease treatment paradigms.

Looking ahead, the future prospects of immunomodulatory nanomaterials are incredibly promising; they are poised to revolutionize healthcare across various domains, including disease treatment, diagnostics, and regenerative medicine. One key area of advancement lies in personalized medicine, where nanomaterials can be tailored to individual patient profiles for more targeted and effective therapies. By integrating biomarkers and imaging modalities, nanomaterial-based platforms can enable early disease detection and personalized treatment strategies, optimize patient outcomes, and minimize side effects. Moreover, immunomodulatory nanomaterials hold immense potential in enhancing the efficacy of existing therapies for complex diseases such as cancer and autoimmune disorders. Nanoparticles and other nanoscale carriers can deliver therapeutic agents directly to disease sites, overcoming biological barriers and enhancing drug bioavailability. This targeted approach not only improves treatment outcomes but also reduces systemic toxicity compared to conventional therapies. Future developments in nanomaterial design, such as stimuli-responsive systems that release drugs in response to specific cues within the body, promise to further optimize therapeutic efficacy and patient compliance. In the realm of infectious diseases, immunomodulatory nanomaterials offer innovative solutions for combating drug-resistant pathogens and emerging infectious threats. Nanoparticle-based vaccines, for instance, can stimulate robust immune responses against viral and bacterial infections, potentially offering broader protection and longer-lasting immunity compared to traditional vaccines. Additionally, nanomaterials can be engineered to deliver antiviral agents directly to infected cells, minimizing viral replication and accelerating recovery rates. As infectious diseases continue to evolve, the adaptability and versatility of nanomaterials provide a critical platform for developing rapid-response therapies and vaccines. Furthermore, the future of immunomodulatory nanomaterials extends into regenerative medicine, where they play a pivotal role in tissue engineering and wound healing. Nanomaterials can facilitate the targeted delivery of growth factors, stem cells, and extracellular matrix components to promote tissue regeneration and repair. By modulating immune responses at the site of injury or disease, nanomaterials can create a favorable microenvironment for tissue remodeling and functional recovery. Advances in nanotechnology are expected to drive innovations in scaffold design, biomimetic materials, and bioactive coatings, offering new solutions for chronic wounds, organ transplantation, and degenerative diseases. Overall, while challenges remain, ongoing research and technological advancements continue to drive the development of immunomodulatory nanomaterials toward safer, more effective, and personalized treatments across a spectrum of diseases, promising a brighter future for patient care and outcomes.

## 8. Review Limitations

This review provides a comprehensive overview of immunomodulatory nanomaterials and their biomedical applications; several aspects of nanomaterial–immune cell interactions fall outside the scope of this work and represent important areas for future investigation. First, the systematic role of physicochemical parameters such as particle size, shape, and surface chemistry in determining immunomodulatory outcomes has not been addressed in depth, as these are covered in dedicated reviews [259,260]. Second, the protein corona—and its dynamic formation, composition, and downstream immune effects—is an emerging area not fully analyzed herein, despite its recognized role in modulating immune recognition and biodistribution. Third, the metabolic fate, degradation pathways, and long-term immunological consequences of nanomaterial persistence in vivo are inadequately understood and were not systematically reviewed. Fourth, this review primarily encompasses in vitro and murine preclinical models; clinical translation outcomes, pharmacokinetics, and patient heterogeneity in immune responses are discussed briefly in the clinical trials section but warrant further dedicated analysis. These limitations underscore opportunities for future systematic and mechanistic investigations.

## 9. Conclusions

Immunomodulatory nanomaterials represent a promising frontier in disease treatment, offering new strategies to enhance or suppress immune responses with precision. By addressing current challenges and leveraging advances in nanotechnology and immunology, these innovative therapies have the potential to revolutionize the treatment of cancer, infectious diseases, autoimmune disorders, and transplant rejection, paving the way for a new era of precision medicine. Different types of nanomaterials utilized in immunomodulation-based disease treatment include lipid-based nanoparticles, polymeric nanoparticles, inorganic nanoparticles, and dendrimers. Lipid-based nanoparticles, such as liposomes, encapsulate drugs or antigens to enhance stability and delivery. Polymeric nanoparticles, made from biodegradable polymers like PLGA, provide controlled release of immunomodulatory agents. Inorganic nanoparticles, including gold and silica, offer unique properties for imaging and therapy. Dendrimers, with their highly branched structures, can carry multiple functional groups for targeted drug delivery, enhancing the precision and efficacy of immunomodulatory treatments. While challenges such as safety concerns, regulatory hurdles, and scalability remain, ongoing advancements in nanotechnology continue to drive innovation and expand the therapeutic landscape. The future of immunomodulatory nanomaterials promises to usher in a new era of personalized medicine, where tailored treatments based on individual patient profiles can optimize outcomes while minimizing adverse effects. With further research and development, these nanomaterials are poised to play a pivotal role in transforming healthcare by offering safer, more effective, and patient-centric approaches to disease treatment and management.

## Figures and Tables

**Table 3:** Interactions of immunomodulatory nanomaterials with different components of the immune system for therapeutic applications.

Nanomaterial Type	Immune System Component	Immune Modulation Type	Disease Treatment Type	Findings	References
Gold nanoparticles	Macrophages	Stimulation	Cancer, allergic diseases, inflammatory diseases	Enhances macrophage activation, increases cytokine production	[60,101,166,167]
Liposomes	Dendritic cells	Both stimulation and suppression	Cancer, autoimmune diseases, infectious diseases	Enhances antigen presentation, increases T-cell activation	[47,168,169]
Polymeric nanoparticles	T-cells, macrophages	Stimulation	Infectious diseases, cancer	Sustained release of antigens, enhances T-cell response, induces effector memory T-cells [CD62L-CD44^+^]	[148,170,171]
Carbon nanotubes	B-cells, monocytes	Stimulation	Cancer, autoimmune diseases	Promotes B-cell proliferation, increases antibody production	[61,62,64]
Dendrimers	Natural killer (NK) cells	Stimulation	Cancer, viral infections	Enhances NK cell activity, increases cytotoxicity against tumor cells	[84,135,138,139]
Magnetic nanoparticles	Macrophages, T-cells	Stimulation	Cancer	Enhances macrophage response	[65,66,67]
Chitosan nanogels	Macrophages	Stimulation	Infectious diseases	Enhances macrophage activity, activates DCs via TLR4-independent cGAS-STING/IFNAR signaling	[83]
Iron oxide nanoparticles	Macrophages, T-cells	Stimulation	Cancer, infectious diseases	Enhances macrophage response, suppresses T-helper 1 cell-mediated immunity, targeted drug delivery	[39,73,74]
Quantum dots	Macrophages, T-cells	Stimulation	Cancer, autoimmune diseases	Enhances macrophage activity, modulates immune tolerance, targeted therapy	[75,76]
Zinc oxide nanoparticles	Natural killer (NK) cells	Stimulation	Cancer, infectious diseases	Antibacterial properties, enhances NK cell cytotoxicity	[77]
Silver nanoparticles/nanorods	Macrophages, dendritic cells	Suppression	Infectious diseases	Antibacterial properties, reduces macrophage-mediated inflammation	[78,79,172]
Mesoporous silica nanoparticles	T-cells, macrophages	Stimulation	Infectious diseases	Enhances T-cell response, induces macrophage polarization	[68,70,71]
PLGA nanoparticles	Macrophages, dendritic cells, T-cells	Stimulation	Cancer, autoimmune diseases	Enhances macrophage activity, activation of DCs, modulation of T-cells	[50,51,52]
Polyethyleneimine nanoparticles	T-cells	Both stimulation and suppression	Cancer	Enhances gene delivery, reduces T-cell-mediated inflammation	[55]

**Table 5:** A representative list of clinical trials in treatment of various diseases using nanomaterials-mediated immunomodulation.

Nanomaterial Type	Immune Modulation	Disease Treatment	Status	Start Date/Year	ClinicalTrials.gov Identifier
Hafnium oxide nanoparticles (NBTXR3)	Stimulation	Head and Neck Squamous Cell Carcinoma, Lung and Liver Metastases	Active, Not Recruiting	16 January 2019	NCT03589339
Gold nanoparticles (C19-A3 GNP peptide)	Suppression	Type 1 Diabetes	Completed	29 September 2016	NCT02837094
Silver nanoparticles	Modulation (Screening)	Lung Inflammatory Disease (Toxicity Screening)	Withdrawn	3 April 2015	NCT02408874
Liposomes (DNA vaccine—VCL-HB01/HM01)	Stimulation	Genital Herpes Simplex Type 2	Completed	December 2013	NCT02030301
Liposomes containing RNA (IVAC_W_bre1_uID)	Stimulation	Triple-Negative Breast Cancer	Completed	October 2016	NCT02316457
Cationic liposomes (JVRS-100)	Stimulation	Relapsed/Refractory Leukemia	Completed	1 March 2009	NCT00860522
Liposomal α-galactosylceramide (RGI-2001)	Suppression	Graft-Versus-Host Disease	Completed	September 2011	NCT01379209
Lipid nanoparticles (mRNA—BNT162b2)	Stimulation	COVID-19 (SARS-CoV-2)	Completed	29 April 2020	NCT04368728
Gold nanoshells (AuroShell)	Stimulation	Prostate Cancer	Completed	February 2016	NCT02680535
Lipid nanoparticles (mRNA-2416, OX40L mRNA)	Stimulation	Relapsed/Refractory Solid Tumors and Lymphoma	Terminated	15 August 2017	NCT03323398
PLGA nanoparticles (CNP-106, tolerogenic)	Suppression	Myasthenia Gravis	Recruiting	30 May 2024	NCT06106672
Iron oxide nanoparticles (Superparamagnetic)	Modulation	Breast Cancer (Sentinel Node Staging)	Completed	01 November 2021	NCT05359783
Iron oxide nanoparticles (NanoTherm)	Stimulation	Glioblastoma Multiforme	Recruiting	January 2024	NCT06271421
Liposomes (CPX-351—Daunorubicin/Cytarabine)	Stimulation	Acute Myeloid Leukemia/Myelofibrosis	Completed	20 February 2019	NCT03878199
LDL-like lipid nanoparticles (Methotrexate-LDE)	Modulation	Atherosclerosis/Coronary Artery Disease	Active (Status Unknown)	10 October 2020	NCT04616872
Gold nanocrystals (CNM-Au8)	Stimulation	Multiple Sclerosis	Completed	19 December 2019	NCT03993171
Iron oxide nanoparticles (Ferumoxytol, USPIO)	Modulation	Multiple Sclerosis (Neuroinflammation Imaging)	Completed	27 November 2015	NCT02511028
Silica nanoparticles (C dots—cRGDY-PEG-Cy5.5)	Modulation	Head and Neck Melanoma (Nodal Mapping)	Active, Not Recruiting	3 April 2014	NCT02106598
Lipid nanoparticles (mRNA—quaratusugene ozeplasmid)	Stimulation	Non-Small Cell Lung Cancer	Recruiting	3 September 2021	NCT04486833
Dendrimers (OP-101, hydroxyl PAMAM)	Suppression	COVID-19 (Hyperinflammation)	Terminated	11 August 2020	NCT04458298
Chitosan nanoparticles	Modulation	Prostate Cancer (AGE Pharmacologic Manipulation)	Terminated	16 January 2019	NCT03712371

## Data Availability

No new data were created or analyzed in this study.
